# Insight into induced charges at metal surfaces and biointerfaces using a polarizable Lennard–Jones potential

**DOI:** 10.1038/s41467-018-03137-8

**Published:** 2018-02-19

**Authors:** Isidro Lorenzo Geada, Hadi Ramezani-Dakhel, Tariq Jamil, Marialore Sulpizi, Hendrik Heinz

**Affiliations:** 1 Department of Physics, University of Mainz, Staudingerweg 7, D-55128 Mainz, Germany; 2 Department of Polymer Engineering, University of Akron, 250S Forge St, Akron, OH 44325 USA; 3 Institute for Molecular Engineering, University of Chicago, 5640 South Ellis Avenue, Chicago, IL 60637 USA; 4 Department of Biochemistry and Molecular Biology, University of Chicago, 929 East 57th Street, Chicago, IL 60637 USA; 5 Department of Chemical and Biological Engineering, University of Colorado-Boulder, 3415 Colorado Ave, Boulder, CO 80309 USA

## Abstract

Metallic nanostructures have become popular for applications in therapeutics, catalysts, imaging, and gene delivery. Molecular dynamics simulations are gaining influence to predict nanostructure assembly and performance; however, instantaneous polarization effects due to induced charges in the free electron gas are not routinely included. Here we present a simple, compatible, and accurate polarizable potential for gold that consists of a Lennard–Jones potential and a harmonically coupled core-shell charge pair for every metal atom. The model reproduces the classical image potential of adsorbed ions as well as surface, bulk, and aqueous interfacial properties in excellent agreement with experiment. Induced charges affect the adsorption of ions onto gold surfaces in the gas phase at a strength similar to chemical bonds while ions and charged peptides in solution are influenced at a strength similar to intermolecular bonds. The proposed model can be applied to complex gold interfaces, electrode processes, and extended to other metals.

## Abstract (web summary)

Molecular dynamics models for predicting the behavior of metallic nanostructures typically do not take into account polarization effects in metals. Here, the authors introduce a polarizable Lennard–Jones potential that provides quantitative insight into the role of induced charges at metal surfaces and related complex material interfaces.

## Introduction

Nanostructures of precious metals find many applications, for example, as catalysts, electrode materials, biomarkers, and therapeutics, including gold (Au) nanorods for photothermal cancer therapy and nanoparticles for gene delivery^1,2^. Metal nano objects have been synthesized in many shapes and sizes, however, control over nucleation, growth, and ligand interactions for nanoscale assembly remains a challenge^3–6^. Understanding the selective synthesis and structure-property relationships requires tremendous efforts by imaging, spectroscopy, and other laboratory techniques^7,8^.

Understanding and discovery can be accelerated by use of atomistic simulations up to the large nanometer scale (e.g., 100 nm) in comparison with experiment^9–15^. The Interface force field (IFF), for example, contains Lennard–Jones parameters for face-centered cubic (fcc) metals to simulate bulk solids, aqueous interfaces, and multiphase materials with polymers and biomacromolecules^9,10^. The parameters reproduce the density, surface tension, and anisotropy of surface energies of (h k l) facets, as well as the mechanical properties in excellent agreement with experiments, even better than some DFT methods^16^. Simulations using this non-polarizable model have proven helpful in understanding the adsorption mechanisms of biomolecules, as well as growth mechanisms and shape preferences of metal nanostructures using particular ligands^13,17–23^. Simulations achieved quantitative agreement with experimental observations^5,13,19,22–25^ yet mainly focused on ligands of low polarity, simple shapes, and simple surface assemblies without accounting for the effects of induced charges and external potentials. It is a shortcoming that the non-polarizable potential does not account for the contribution of the induced charges to interfacial processes during Molecular Dynamics (MD) or Monte Carlo (MC) simulations. Polarization on the metal surface was shown to affect the surface adsorption of molecules and could only be “added” a posteriori^26^. Such a posteriori calculations are significantly less accurate and impractical as they are uncoupled from the dynamics, require time-consuming post-processing of simulation outputs (coordinates and energies), and cannot be applied to corrugated metal surfaces or under external electric fields. The effects of induced charges in the metal are known to be substantial in vacuum^27–29^, under external potentials in electrodes^30,31^, in the presence of ionic liquids^21^, and at high ionic strength in solution^26^, although expected to be weaker in dilute aqueous solution^26^.

Attempts to include polarization have also been made in alternative models for metallic nanostructures and electrodes. The GolP force field adds permanent dipoles to every atom to account for effects of induced charges^11,12,32,33^. The dipoles are implemented as fixed rods and shift the image plane for positively charged vs. negatively charged species on the metal surface. Another limitation is that surface energies and mechanical properties of the metal have not been reproduced and the compatibility with biomolecular force fields requires many adjustable parameters^12^. Several further models have also been developed to describe metallic electrodes at constant potential^34–39^. Siepmann and Sprik^34^ pioneered models under a constant applied potential in which variable charges are added to the electrode and their magnitude is adjusted on-the-fly according to a variational procedure. The model matches image potentials and accounts for the polarization of the electrode by the electrolyte. However, parameters for the charge distribution are then necessary and molecular dynamics simulations require on-the-fly adjustments of the charge distribution. Moreover, surface energies, as well as interfacial energies of the metals have not been validated relative to experimental data and the original energy expressions are difficult to use due to their complexity (see Supplementary Note 1 for details).

**Fig. 1:**
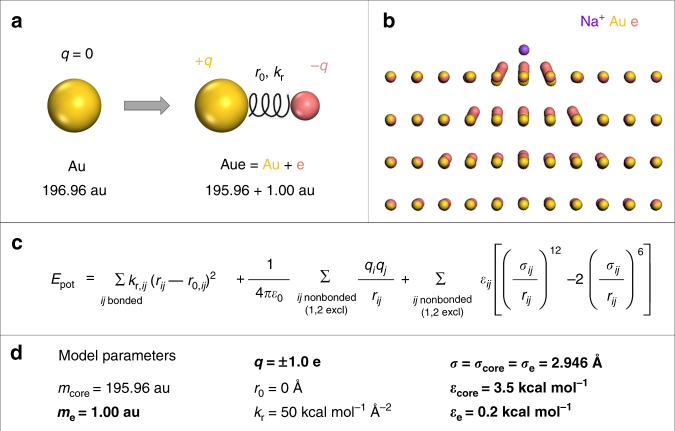
Polarizable Lennard–Jones model for gold. **a** Extension of the simple Lennard–Jones model with dummy electrons to add features of the free electron gas. The virtual electrons rest at the atom core and carry a mass of 1 au. **b** Visualization of the dummy electrons on the Au (111) surface in the presence of an adsorbed sodium ion in vacuum. The induced charges spread across several atomic layers laterally and beneath the top atomic layer. **c** The energy expression contains terms for harmonic bond stretching, Coulomb energy, and van-der-Waals energy (Lennard–Jones potential). **d** The model uses five independent parameters (highlighted in bold) including the mass of the dummy electron *m*_e_, a combination of the charge *q* and the bond stretching constant *k*_r_, whereby a certain ratio *α* = *q*^2^/(2*k*_r_) determines the magnitude of the image potential, as well as the Lennard–Jones parameters *σ*, *ε*_core_, and *ε*_e_. The total mass of the gold atom (*m*_core_ + *m*_e_) and the rest position of the dummy electron *r*_0_ = 0 Å are constants

Here we introduce a simple polarizable Lennard–Jones model for metallic gold. It adds the correct amount of attractive polarization to the neat Lennard–Jones potential and retains the mobility of all atoms, as well as the other advantageous aspects of the nonpolarizable model (Fig. 1a)^10^. The model reproduces the classical image potential, lattice parameters, surface energy, and hydration energy with water in excellent agreement with experiments, and a good correlation with results from density functional theory is demonstrated. The model is compatible with common biomolecular and materials-oriented force fields (AMBER, CHARMM, CVFF, DREIDING, GROMACS, IFF, OPLS-AA) and does not require additional parameters to simulate interfaces with solvents and biomolecules as it follows the IFF approach^9,24^. The proposed model facilitates new insights into the interaction of metal surfaces with ions, solvents, biological and polymeric ligands in solution, including atomically resolved details of molecular recognition on (h k l) surfaces and nanocrystals, binding energies, and assembly preferences of metal nanostructures into superstructures. The image potential is found to strongly influence the adsorption of isolated ions onto gold surfaces in the gas phase, comparable to chemical bonds, while polarization effects in the condensed phase are an order of magnitude weaker due to electrostatic screening interactions, i.e., due to close proximity of mutually compensating charges. The loss in polarization at metal interfaces in the condensed state is a general result for metal-oxide and metal-solution interfaces. However, polarization effects remain significant and comparable to the strength of intermolecular bonds. For example, the relative contributions of electrostatic vs. van-der-Waals energy to adsorption are significantly affected by polarization. Ions in solution and ionic groups in peptides change their equilibrium distance and interaction energy with the metal surface in response to polarization. The extent of this effect depends on the type of ion and (hkl) facet. Overall, polarization enhances the attraction of ionic peptides by ~10%. Adsorption of ionic peptides onto gold surfaces involves (hkl) facet-specific soft epitaxial interactions, complex conformational equilibria, and internal salt bridges. The on-the-fly inclusion of induced charges in atomistic simulations allows more accurate simulations of complex, reconfigurable, and bioprogrammable metal nanostructures, including electrocatalysts^40^, complexes and superlattices with large proteins, lipids, and DNA^41–43^. The reliability of simulation results in the presence of electrically charged ligands, solvents, and under external electric fields is significantly increased, and the model is extensible to other metals. Sample surface models and force field files are provided in the Supplementary Data set 1.

## Results

### Formulation of the core-electron polarizable model

The purpose of this classical polarizable model is (1) to represent electron density around atomic centers that can flexibly respond to the presence of external charges, (2) to achieve broad compatibility of the potential, as well as (3) to retain the advantages of the simple, non-polarizable Lennard–Jones model^10^. The first goal is achieved by reproducing the image charge potential induced by an external charge in proximity to the metal surface (Supplementary Fig. 1a)^26,28^. The reference for induced charges is the image plane located at the jellium edge. The jellium edge is positioned one-half lattice spacing atop the outermost atomic layer of a metal surface and approximately represents the location of the effective metal surface (Supplementary Fig. 1a–d)^26,28,44^. We describe the resulting attraction using a pair of a positive core charge +*q* and a virtual (dummy) electron of negative charge −*q* that are located at the center of each metal atom and coupled by a harmonic spring (Fig. 1a, b). The virtual electron represents valence electron density that can be polarized and aims at capturing some properties of a free electron gas. In the presence of an external charge, the dummy electrons leave the center position on the metal atoms and create an oriented attractive dipole. In the absence of an external charge, the dummy electrons rest at the atomic core without any dipoles created. The remaining difference to a continuum free electron gas is that the virtual electrons in the classical model are tethered to the atom cores and cannot freely travel inside the metal (see Supplementary Notes 2, 3, and 4).

**Fig. 2:**
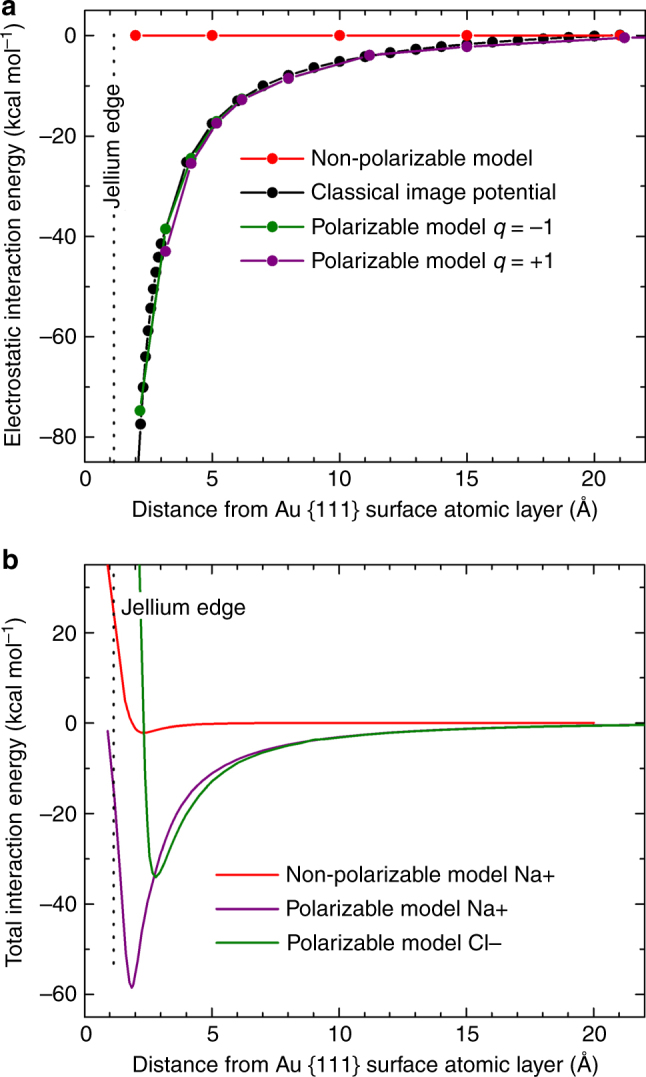
Interaction of a positive charge (sodium ion) and a negative charge (chloride ion) with the metal surface in vacuum. **a** The electrostatic interaction energy as a function of distance from the surface using energy minimization. The polarizable model closely approximates the classical image potential and shows a large improvement over the non-polarizable Lennard–Jones potential. The curve remains the same for any cation or anion with a charge of +1.0e or −1.0e regardless of chemical identity. **b** The total energy of interaction of a sodium ion and a chloride ion with the Au (111) surface as a function of distance in molecular dynamics simulation at 298 K. Stable minima are seen near 1.86 Å and 2.81 Å distance from the surface atoms, respectively. The presence of Coulomb and Lennard–Jones interactions in the polarizable model imposes a barrier to dissolution in the metal of >50 kcal mol^−1^. The non-polarizable model does not reproduce the strong attraction. Computations involved 3D periodic simulation boxes and common Ewald summation of Coulomb interactions. A box size of at least 10 × 10 × 100 nm^3^ is recommended to reduce errors in the computed image charge potential due to long-range interactions to <1%

The second aim of broad compatibility is realized by using the same energy function as in common biomolecular and materials-oriented force fields (AMBER, CHARMM, CVFF, GROMACS, IFF, OPLS-AA) and following the IFF protocol for thermodynamic consistency^9^. The energy expression for the polarizable model contains terms for harmonic bond stretching, Coulomb interactions, and a 12–6 Lennard–Jones potential (Fig. 1c). Following standard rules in the above force fields, nonbond interactions between 1, 2 bonded atom cores (+*q*) and virtual electrons (−*q*) on the same gold atoms are excluded in energy and in force calculations during molecular dynamics simulation. A small mass of the dummy electron (1 au) relative to atom core (196 au) was chosen to enable much faster movement of the virtual electron and avoid interference with the motion of the atomic centers. This is a necessary condition for the function of the model, analogous to the Born-Oppenheimer approximation (see Supplementary Note 3)^45^. The charges *q* and the bond stretching constant *k*_r_ represent the polarizability of the gold atoms *α* in the model and are interrelated as *α* = *q*^2^*/(*2*k*_r_) (see Supplementary Note 5). The charge *q* is +1.0e on the atom core, −1.0e on the dummy electron, and the spring constant *k*_*r*_ equals 50 kcal mol^−1^ Å^−2^ (Fig. 1d). These settings result in displacements of the dummy electrons in a range of 0.0–0.8 Å that is smaller than the atomic radius of gold and reproduce the image potential (Fig. 2a). *k*_r_ was chosen large enough to reduce random (thermal) motion of the dummy electrons. Higher charges were avoided due to potential failure modes by recombination with oppositely charged ions. Besides, the precise choice of *q* and *k*_r_ has minor impacts on the model as long as the polarizability *q*^2^/(2*k*_r_) is constant to yield the same displacement of dummy electrons under an applied potential, and thus the same image potential.

The Lennard–Jones parameters *σ* and *ε* for the atom core and for the dummy electron are of equal importance, whereby *σ* mainly reproduces the lattice parameter of gold and the values of *ε* mainly determines the value of the surface energy. *ε*_e_ also stabilizes the dummy electrons. The assignment of Lennard–Jones parameters for the atom cores and for the dummy electrons is necessary to maintain the stability of metal nanostructures and their surfaces for the chosen values ±*q* and *k*_r_ up to high temperatures >1000 K. Atom cores and virtual electrons share identical *σ* values as they occupy the same van-der-Waals volume (Fig. 1d). This setting prevents possible failure modes such as spontaneous polarization and recombination of dummy electrons with neighbor atom cores, as well as with highly charged cations like Ca^2+^ near the gold surface (see Supplementary Note 2). Overall, the model has elements of a Lorentz–Drude oscillator^46,47^ and earlier core-shell models^48,49^. It is, however, distinct from these models since all energy terms are necessary for the function, including the Lennard–Jones potential between all non-bonded nuclei and virtual electrons (Fig. 1c and Supplementary Note 1).

The third aim of retaining the advantages of the nonpolarizable Lennard-Jones parameters^10^ is fulfilled by following the IFF approach^9^. The individual atoms, consisting of atom core and coupled dummy electron, serve as individual units to assemble models of nanostructures and alloys just as the simple atoms using the nonpolarizable Lennard–Jones potential^10,20,24^. The validation of structural, interfacial, and mechanical properties shows equal or improved performance of the polarizable model compared to the non-polarizable model relative to experiment (Table 1 and Supplementary Table 1). The performance is also better than DFT methods.

**Table 1:** Computed bulk and interfacial properties of gold using the new polarizable potential in comparison to experimental data. Results from DFT calculations are also shown for comparison and deviate up to an order of magnitude more from experiment than the force field

Property	Expt	Ref.	Sim (FF)	Dev.	Sim (DFT)^a^	Dev.
Density (g cm^−3^)	19.288	^52^	19.288	0.0%	18.2	−5.6%
Au (111) surface energy (J m^−2^)	1.54	^88^	1.55	+0.6%	0.74	−52%
Au-water monolayer hydration energy (kcal mol^−1^)	13; 15.5	^58, 59^	~13.5	Agrees	NA	
Image charge potential	~1/*r*^b^	^28, 76^	match		no match	
Au-water interface tension (J m^−2^)	<1.47	^9, 89^ ^,c^	1.23	Agrees	NA	
Bulk modulus (GPa)	173	^52^	145	−16%	140	−19%

^a^Using the PBE functional. Similar deviations are also observed with other density functionals (ref.^16^). The simulation of hydration energies and interfacial tensions by ab-initio dynamics is difficult due to limitations in time scale (>100 ps using IFF)

^b^The image charge potential of a single ion in vacuum is inversely proportional to the distance *r* from the metal surface

^c^Experimental data for the Au-water interfacial tension are based on a contact angle of 0° and the Young equation and provide an upper limit, not an exact value (refs.^10, 89^). The computed Au-water interfacial tension of 1.23 J m^−2^ corresponds to an Au-water interfacial energy of 1.17 J m^−2^ and an entropy contribution of +0.06 J m^−2^ as water molecules partially lose mobility upon adsorption (see ref.^10^)

**Fig. 3:**
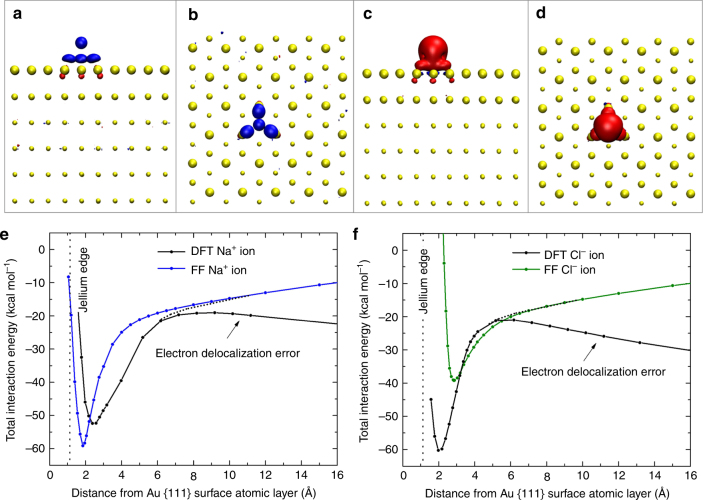
Equilibrium position and total interaction energy of sodium and chloride ions on gold (111) surfaces in DFT calculations and with the polarizable force field. **a**, **b** Na^+^ ions are preferably located above epitaxial (hollow) sites in DFT calculations and in calculations with the polarizable FF. The contour of the electron density difference upon binding shows induced negative charge (blue) atop the surface atoms, similar to positions of dummy atoms (Fig. 1b) (contour level 0.02e Å^−3^). **c**, **d** Cl^−^ ions preferably bind to epitaxial (hollow) sites, too, in DFT calculations and with the polarizable FF. The contour of the electron density difference upon binding shows induced positive charge (dark red) atop the surface atoms (contour level 0.02e Å^−3^) and some negative charge in the plane of surface atoms. **e**, **f** Total interaction energies of Na^+^ and Cl^−^ ions. Similar curves are seen for sodium ions (**e**) in DFT calculations and with the polarizable FF. Differences are noted for chloride (**f**). The larger chloride ion approaches the Au surface very closely in DFT, possibly due to chemisorption, which is neglected with the force field. DFT does not reproduce long-range features (>5 Å distance) of the image potential that decay as 1/*r* with distance *r*. Therefore, the vertical position of the DFT curve remains uncertain. Small identical simulation boxes of 1.44 × 1.50 × 10.0 nm^3^ and a series of energy minimizations were employed to obtain both types of data

An open question remains the exact static average electric dipole polarizability of gold atoms as experimental reference data are not available. The polarizability of Au atoms in the gas phase can be analytically expressed in the model as *α* = *q*^2^*/(*2*k*_r_), or $\alpha _V = q^2/(8\pi \varepsilon _0k_{\mathrm{r}})$, and amounts to *α*_*V*_ = 3.3 Å^3^ (see Supplementary Note 5 and Supplementary Fig. 2). High level quantum mechanical calculations such as CASPT2^50^ and QCISD(T)^51^ suggest *α*_*V*_ to be 4.1 and 5.3 Å^3^ with an uncertainty exceeding 1 Å^3^. The polarizability *α*_*V*_ in the classical model tends to be at the lower end while much improved over the non-polarizable model (*α*_*V*_ = 0.0 Å^3^). In principle, polarization in the classical model can be validated using the image potential or the atomic polarizability as a reference. We chose the image potential because the exact location of the image plane is known from measurements of surface state energies (essentially at the jellium edge for gold)^44^, and this approach can be applied to all metals^26–28^. In contrast, reliable reference data for atomic polarizabilities of metals remain hard to come by^50,51^. The comparison of ion attraction using the classical polarizable model and DFT calculations (Fig. 3) also indicates that the amount of polarization in the classical polarizable model is about right (see details in Supplementary Note 3).

In summary, the polarizable model includes five independent parameters, namely, the mass of the dummy electron (set to 1 au), the ratio *q*^2^/*k*_r_, *σ*, *ε*_core_, and *ε*_e_. The parameter space was explored in over 500 calculations to test the robustness of the model, including charges from ± 0.0e to ± 2.0e, *k*_r_ values from 0 to 200 kcal mol^−1^ Å^−2^, *σ* values from 0 to 5 Å, as well as *ε* values from 0 to 8 kcal mol^−1^ for atomic nuclei and virtual electrons, respectively. The parameters of the final model were obtained using a least squares fit to reproduce the classical image potential, the metal lattice parameters, the (111) surface energy, and maintaining a physical rationale (see Table 1 and Supplementary Note 2)^10^. The polarizable potential features full atom mobility and compatibility with parameters for water and biomolecules, just as the prior Lennard–Jones potential^10,18–21,26^, an accurate representation of induced charges (Fig. 2) and improvements in mechanical properties (Supplementary Table 1).

### Adsorption of cations and anions in vacuum

The simulation of the electrostatic interaction energy of a sodium ion with the Au (111) surface shows a near-quantitative fit with the continuum image potential, which demonstrates the incorporation of attractive polarization in the model (Fig. 2a, purple line). The results are the same for Li^+^, K^+^, and any other cations with a charge of +1.0e as the chemical identity of the ions is not important for the electrostatic (Coulomb-only) potential.

The continuum image potential is known to be a good approximation for distances larger than 2.5 Å from the image plane^27,44^. At shorter distances, repulsion from the metal surface needs to be taken into account (Fig. 2), which in the model is composed of an indirect contribution by the bond energy of the virtual electrons in the metal, as well as a direct contribution by the Lennard–Jones potential on the atom cores and on the dummy electrons (Supplementary Fig. 3). The total interaction energy of sodium ions has a minimum of −50 kcal mol^−1^ (−2.1 eV) at a distance of ~1.9 Å from the metal surface atomic layer, similar in strength to a chemical bond (Fig. 2b). The difference to a non-polarizable model is very large. The total interaction energy is then only −2 kcal mol^−1^ (−0.1 eV) because electrostatic attraction is neglected. The polarizable model achieves improvements greater than an order of magnitude.

The adherence of the computed polarization energy to the classical image potential was also tested for negatively charged ions such as Cl^−^ ions, which are representative of F^−^, Br^−^, I^−^, OH^−^, and other anions. The electrostatic interaction profile is about equally close as for positively charged ions (Fig. 2a, green line). At ~2 Å distance from the jellium edge for positive charges and at about 1 Å distance for negative charges, respectively, the computed electrostatic energy rapidly approaches large negative values that are balanced by the bond stretching potential of the virtual electrons and the repulsive Lennard–Jones potential to yield the total interaction energy. As the Lennard–Jones potential is ion-specific, the total interaction energy also becomes ion-specific at distances below ~5 Å distance from the jellium edge (Fig. 2b and Supplementary Note 6 for details). A large improvement in comparison to the non-polarizable model is seen, and details of the individual energy contributions including bond stretching are shown in Supplementary Fig. 3.

Electrical monopoles from single ions cause significant long-range interactions, and convergent computations of the electrostatic attraction to the metal surface in vacuum require sufficiently large 3D periodic boxes with recommended dimensions of at least 10 × 10 × 100 nm^3^. Interactions between the original point charge and its periodic images under periodic boundary conditions are then negligible (<1%). Smaller periodic boxes restrict lateral spreading of the surface-induced charge and add ion-ion repulsion, leading to reduced electrostatic interaction energies with the surface (Supplementary Fig. 4, Supplementary Table 2, and Supplementary Note 7).

It is also possible to combine the polarizable model with the non-polarizable model. We tested how many layers of polarizable atoms vs. non-polarizable atoms suffice to capture the image potential (Supplementary Fig. 5). As expected, best results are obtained when dummy electrons are included on all atoms. The total interaction energy of ions nevertheless shows that already one layer of dummy electrons adds 70% of the total polarization energy, and the inclusion of one or two polarizable atomic layers provides a great improvement over the non-polarizable model (Supplementary Fig. 5).

The comparison of ion adsorption using the polarizable force field and Density Functional Theory (DFT), performed at the PBE level with Grimme D3 corrections, provides further insight and validation (Fig. 3). Energy minimization shows that sodium ions and chloride ions preferentially adsorb onto epitaxial (hollow) sites rather than top sites (Fig. 3a–d). The polarizable force field, using smaller identical simulation boxes (1.44 × 1.50 × 10 nm^3^), shows the same preferences and therefore produces energy landscapes consistent with DFT calculations^18,21^.

DFT calculations also show the induction of a negative image charge below the top layer atoms for Na^+^ ions (Fig. 3a, b), as well as of a positive induced charge in the surface plane of metal atoms for Cl^−^ ions (Fig. 3c, d). The image charge distributes laterally and vertically up to several atomic layers, which can be seen by Friedel oscillations in a complete quantum mechanical analysis (Supplementary Fig. 1b–d)^28^. The classical polarizable model shows the displacement of the dummy electrons out of equilibrium positions as a response, which assumes similar lateral and vertical dimensions as the Friedel oscillations (Fig. 1b, Supplementary Fig. 1e, f, and Supplementary Note 3).

The interaction energies of sodium ions and chloride ions with the (111) surface as a function of distance are in reasonable agreement using the polarizable force field (blue and green lines) and DFT calculations (black lines), even though no DFT data was used in the derivation of the force field parameters (Fig. 3e, f). In the short range, the adsorption curves are nearly the same for Na^+^ ions while adsorption of chloride ions is somewhat stronger using DFT. The force field suggests a larger equilibrium distance of Cl^−^ relative to Na^+^ due its larger ionic radius^52^, while DFT calculations show a shorter equilibrium distance that may be related to partially covalent bonding of Cl^−^ ions to Au. All energy contributions in the force field are shown in Supplementary Fig. 6 (see also Supplementary Note 6). On the other hand, DFT does not reproduce the physically expected 1/*r* convergence of the image charge potential to zero in the long range. This is a well-known problem of GGA density functionals (therefore also of the PBE functional) related to a strong electron delocalization error and an inaccurate description of the long range van-der-Waals interaction^53,54^. As a result, the vertical position of the DFT curve remains somewhat uncertain. Figure 3e, f shows likely fits, assuming the near-part of the DFT to be correct, whereby the DFT adsorption energies could also be up to about 10 kcal mol^−1^ closer towards zero. Overall, the force field reproduces the energy landscape and the physically justified portion of the DFT data. The data also suggest that the classical image potential is a suitable reference for the parameterization of polarization energies.

### Gold-water interfaces and adsorption of ions in solution

**Fig. 4:**
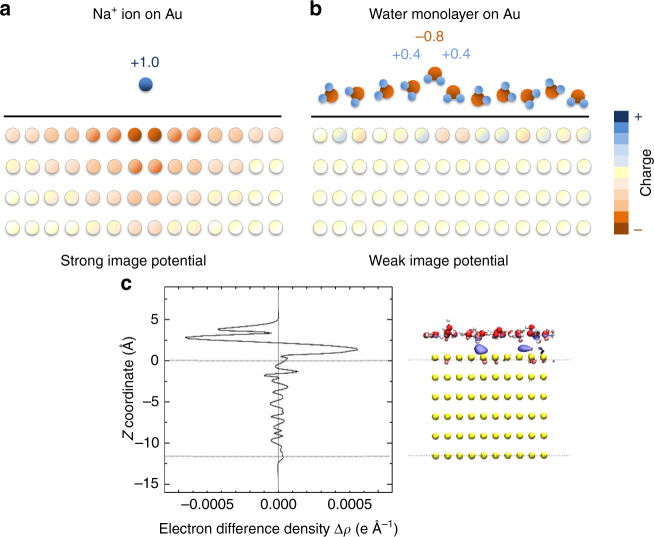
Origin of major differences in the magnitude of the image potential for isolated ions in the gas phase vs. charge-neutral molecules on gold surfaces. **a** An isolated ion with a net charge of +1.0e in the gas phase induces significant charges in the metal surface that spread across several atomic layers vertically and several nanometers laterally. **b** In contrast, neutral water molecules in the condensed phase contain dipoles with opposite charges in close proximity, unable to induce a long-range pattern of image charges. Electrostatic interactions are screened and the image potential is more than order of magnitude smaller. The reduction in image potential in (**b**) also applies for metal-oxide and metal-ceramic interfaces. **c** Electron density difference at the interface of a water monolayer with an Au (111) surface according to DFT (blue = negative charge, isovalue −0.027e Å^−3^, red = positive charge, isovalue +0.027e Å^−3^). Induced charges are small, illustrated by visualization on the right hand side showing some negative induced charge in the vicinity of positively charged hydrogen atoms of water molecules close to the gold surface (blue = negative charge difference, red = positive charge difference)

**Fig. 5:**
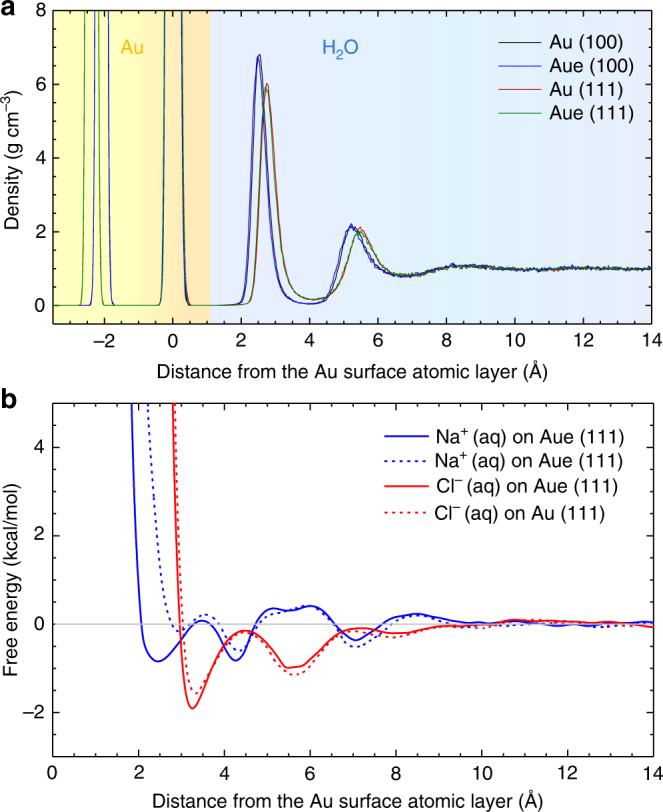
Density profile of water and free energy profiles of dissolved ions on the gold surface. **a** Computed density profile of water on the Au (111) and Au (100) surfaces with and without polarization. The difference between the polarizable and the nonpolarizable model is barely visible. Two distinct and two further weak surface layers of water are seen on both surfaces. Water molecules approach the top layer of the metal surface atoms 0.25 Å more closely on the (100) surface than on the (111) surface. **b** Computed free energy profile of sodium and chloride ions on the Au (111) surface in water with and without polarization. Polarization significantly changes preferred distances and adsorption energies of the ions, which is particularly visible for sodium ions at ~2.5 Å distance. Chloride ions exhibit different preferred distances compared to sodium ions and are more strongly attracted, enhanced at ~3.3 Å distance due to polarization. Adsorption energies are on the order of van-der-Waals contacts and weak hydrogen bonds. The zero point of the *z* coordinate corresponds to the position of the top layer of gold surface atoms in (**a**) and (**b**)

The gold (111) and gold (100) interfaces with water and ions provide further validation of the model and new insights into the interfacial dynamics (Table 2, Figs 4 and 5). Water molecules were represented by a common flexible, non-polarizable SPC/E model, and similar water models such as TIP3P can equally be used^55^. Computed adsorption energies of individual water molecules^56–58^, molecular monolayers of water^58,59^, interfacial tensions^10,60^, and hydration energies are in excellent agreement with experimental data (Table 2 and Supplementary Note 8). The deviation in monolayer adsorption energy is only ~5%. A difference between the polarizable and the non-polarizable force field (where the gold is described by simple Lennard–Jones parameters^10^) is seen for the adsorption of single water molecules while the models perform de facto the same for bulk water. The reason for minor differences in binding energies in solution compared to large differences in vacuum are the short multipole lengths on the order of ~1 Å in neutral molecules that are equal to the length of chemical bonds. These lengths are more than one order of magnitude smaller than the distance between monopoles in vacuum of >50 Å that is required to fully develop unperturbed image charges (Fig. 4a, b). Image charges are therefore of limited relevance for flat metal surfaces in aqueous solution as the multipoles within neutral molecules and the dipoles of neighbor molecules screen the partial charges.

**Table 2:** Detailed properties of gold-water interfaces according to computation with the polarizable force field in comparison to the nonpolarizable force field (Lennard–Jones only model) and experimental data

Gold surface model	ΔH_ads_ of a single water molecule from the gas phase (kcal mol^−1^)	ΔH_ads_ of a water monolayer from the gas phase (kcal mol^−1^)	Solid-liquid interfacial energy (mJ m^−2^)	Hydration energy (mJ m^−2^)
Au (100)	−3.9 ± 0.5	−14.3 ± 0.1	1131 ± 2	−470 ± 2
Aue (100)	−5.5 ± 0.5	−14.3 ± 0.1	1153 ± 2	−474 ± 2
Au (111)	−5.8 ± 0.5	−12.8 ± 0.1	1148 ± 2	−387 ± 2
Aue (111)	−6.1 ± 0.5	−13.0 ± 0.1	1169 ± 2	−389 ± 2
Expt (polycryst.)	≈−5 (physisorbed)^a^	−13; −15.5^b^	<1410 (wetting)^c^	

Source: ^56–59^

^a^Ref.^56^ (−25 to −35 kcal mol^−1^ are observed for chemisorbed water)

^b^Refs.^58,59^

^c^Refs.^10,89^

The difference in the electron density on the Au (111) surface upon binding of a single molecular layer of water was also analyzed by DFT calculations (Fig. 4c). The electron density was obtained as the difference between the electron density of the Au (111)-water monolayer interface, the single surface, and the single water monolayer. The presence of only small negative (blue) and positive (red) charge shifts near the surface can be seen, which are much less significant than electron density differences at the interface with single ions in vacuum (Fig. 3a–d and Supplementary Fig. 1)^26,50^. Correlations between the in-plane structure of the liquid and the in-plane structure of the charge distribution in the metal can also be seen whereby individual snapshots (Fig. 4c) indicate a more detailed structure than time-averaged trajectories in classical molecular dynamics (Supplementary Movies 1 to 8). Earlier studies have also suggested highly collective behavior of water on metal surfaces and fluctuations over large length scales and time scales (see additional references in Supplementary Note 8)^10,26,61^.

Overall, the new polarizable, as well as the nonpolarizable model^10^ are quantitative models for metal-water interfaces due to the excellent match of computed hydration energies with experimental data, which is documented here for the first time (Table 2). Previously, available experimental data for hydration energies were not compared with computed values (only upper limits of the metal-water interfacial energies that are accessible from contact angle measurements and Young’s equation were compared, see Supplementary Note 8)^10^.

Since differences in hydration energy with and without polarization in the condensed phase are small, the density profiles of pure bulk water are nearly identical using the polarizable vs. the nonpolarizable model (Fig. 5a). The curve including polarization is ~0.05 Å closer to the metal surface. The sharp peak at about 2.5 Å distance from the top atomic layer of gold (111) and (100) surfaces corresponds to the first adsorbed layer of water and indicates strong metal-water interactions. It is followed by a weaker second peak at 5 to 5.5 Å, and a less visible third peak near 8.5 Å. The trend is coupled with slightly more negative hydration energies for the polarizable model (Table 2).

More significant differences are noticed between the (100) surface and the (111) surface. Water molecules approach the outermost atomic layer on the (100) surface ~0.25 Å more closely (Fig. 5a), and therefore cause more attractive polarization for single water molecules as well (Table 2). The origin of these differences is the wider quadratic surface pattern (L2 spacing) on (100) surfaces compared to a narrower hexagonal surface pattern (L1 spacing) on (111) surfaces (see Supplementary Movies 1 to 8)^18^. Overall, the effect of induced charges on the adsorption of pure bulk water is negligible so that a non-polarizable model performs the same (Fig. 4b).

The attraction of ions to the gold surface in water, however, indicates significant differences in preferred distances and free energy profiles when using the polarizable model vs. the nonpolarizable model (Fig. 5b). The overall attraction of the ions to the surface is comparatively weak, less than −2 kcal mol^−1^ at any distance, owed to the hydration shells and similar polarity of water and cations. Sodium ions prefer some direct contact with the gold surface at ~2.5 Å distance in the polarizable model, which corresponds to the closest distance of water molecules, and have no significant attraction in the non-polarizable model (Fig. 5a,b). Larger chloride ions remain further away from the surface at ~3.25 Å distance and exhibit increased adsorption of −1.9 kcal mol^−1^ in the polarizable model vs. −1.5 kcal mol^−1^ in the nonpolarizable model. The preferred distances of sodium and chloride ions from the surface are quite different from each other (Fig. 5b), some within the density maxima of water layers and others outside the dense water layers (Fig. 5a). A large difference is that sodium ions approach the Au surface 0.5–0.7 Å more closely in the polarizable model than in the non-polarizable model. The attraction at distances larger than ~5 Å tends to be weaker in the polarizable model compared to the non-polarizable model. Stronger attraction of chloride ions vs. sodium ions onto gold surfaces is consistent with measurements of negative zeta potentials in the presence of chloride ions^62^. The models clearly show that the adsorption of ions to the metal surface depends on the type of ions and on contributions by polarization, and these effects are not negligible. For example, specific differences in the adsorption of ions are used to direct the growth of metal nanocrystals^2,3,63,64^. The polarizable model can provide quantitative visualizations and energies to explain and further utilize such effects.

### Influence of polarization on the adsorption of charged peptides

**Fig. 6:**
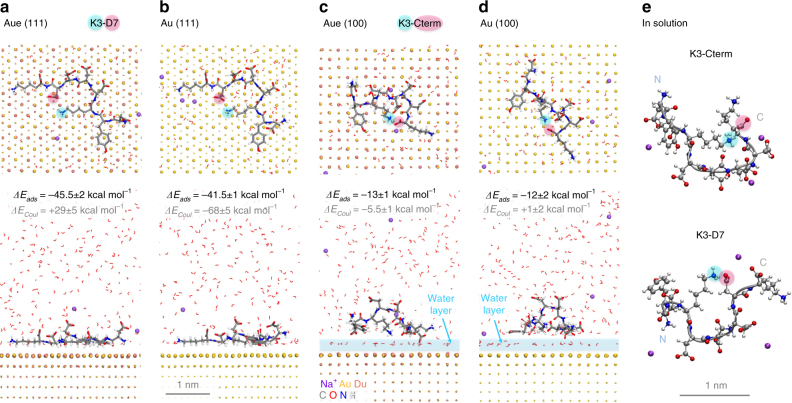
Representative conformations and adsorption energies of an ionic peptide on gold surfaces. The interaction of the peptide DYKDDDDK (FLAG-Tag) with gold (111) and (100) surfaces was analyzed in aqueous solution at 298 K and pH 7 (FLAG-Na3) using the polarizable model (Aue) and the nonpolarizable model (Au).  **a**, **b**. On the (111) surface, the peptide assumes a flat-on conformation in direct contact with the surface using both models. The preferred peptide conformation is very similar and involves a K3-D7 salt bridge (highlighted). Adsorption is driven by soft epitaxy, i.e., proximity of polarizable atoms (C, O, N) to hollow sites in the 2nd and 3rd subsurface layer, and avoidance of atoms in the top layer. Overall adsorption is somewhat stronger using the polarizable model and the contribution of Coulomb energy to adsorption is of opposite sign (see text). **c**,** d** On the (100) surface, the peptide maintains a distance of one water layer from the Au surface atoms (see highlight) and retains conformational flexibility similar to that in solution. The preferred adsorbed conformation involves an ion pair between K3 and the C terminus. Adsorption energies are about the same for polarizable and non-polarizable models and much weaker in comparison to the (111) surface. Coulomb contributions to the adsorption energy differ significantly between the two models. **e** Preferred conformations of the FLAG-tag peptide in solution. The K3-Cterm salt bridge corresponds to lowest energy, while also K3-D7 bridges, Nterm-D7, D4-K8, Nterm-D6, Nterm-D5, and D6-K8 bridges were temporarily observed. Water molecules are partially shown in panels (**a**–**d**). The three sodium ions per peptide often move several nanometers away from the peptide and are shown only partly in panels (**a**) and (**b**)

The adsorption of a highly ionic peptide on gold (111) and (100) surfaces in aqueous solution was investigated to further evaluate the influence of induced charges on biomolecular interfaces of gold (Fig. 6). The common FLAG peptide tag^65^ DYKDDDDK carries multiple charges [NH_3_(+)-D(−)YK(+)D(−)D(−)D(−)D(−)K(+)-CO_2_(−) · 3 Na(+)] and has been studied experimentally on gold surfaces^19,66^. Representative conformations on the (111) and (100) surfaces in aqueous solution at 298 K and pH 7 show only small differences using the polarizable model (Aue) vs. the nonpolarizable model (Au). The peptide undergoes very sensitive conformational equilibria in solution that involve different salt bridges. The salt bridges also determine low energy conformations when adsorbed on the surface and extensive conformation sampling using different start structures was necessary to obtain consistent conformations of low energy (see Supplementary Note 9). Adsorption energies are enhanced when polarizability is included, similar to the strength of a hydrogen bond or several van-der-Waals contacts (1 to 4 kcal mol^−1^). Major changes, however, occur in the magnitude of Coulomb vs. van-der-Waals contributions to adsorption, allowing a more realistic analysis of the binding mechanism (Fig. 6a–d).

Specifically, the peptide remains in direct contact with the (111) surface and assumes a flat-on conformation with soft epitaxial contacts as previously described (Fig. 6a, b)^18,20,28^. Polarizable atoms (C, O, N) prefer proximity to hollow sites in the 2nd and 3rd subsurface atomic layers of gold and avoid contact with atoms in the top layer^20,24^. A conformation with a K3-D7 salt bridge is preferred on the surface while the structure in aqueous solution is dominated by K3-Cterm and a K3-D7 salt bridges (Fig. 6e). Incorporation of polarizability increases the adsorption energy by about 10% (Fig. 6a, b).

On the (100) surface, the FLAG peptide retains conformational flexibility similar to that in solution and maintains a distance of one water layer from the Au surface atoms, resulting in much weaker binding (Fig. 6c, d). The preferred conformation on the surface involves an ion pair between K3 and the C terminus similar to the state in solution (Fig. 6c, d). Adsorption is clearly weaker than on the (111) surface and almost equal for polarizable and non-polarizable models.

Large differences are seen in Coulomb vs. van-der-Waals contributions to the adsorption energy (Fig. 6a–d). Water molecules are bound to the surface via electrostatic interactions in the polarizable, more realistic model, whereas binding is purely of van-der-Waals type in the non-polarizable model^10^. Adsorption of the FLAG peptide onto the (111) surface replaces about 30 surface-bound polar water molecules by amino acids of lesser polarity (Fig. 6a, b). Therefore, Coulomb contributions to adsorption are unfavorable (+29 kcal mol^−1^) and adsorption of the peptide occurs through strong, overcompensating van-der-Waals type epitaxial interactions. In contrast, in the non-polarizable model, the Coulomb contribution dominates peptide adsorption (−68 kcal mol^−1^). Water molecules then bind via van-der-Waals interactions to the gold surface and replacement by the less polar peptide frees up the ~30 surface-bound water molecules that can then fully interact with other water molecules via electrostatic hydrogen bonds. On the (100) surface, in contrast, the first superficial water layer, which accounts for over 80% of gold-water interactions, is less affected by peptide adsorption (Fig. 6c, d). The Coulomb contributions to peptide binding are slightly stronger for the polarizable model (−5 kcal mol^−1^) compared to the non-polarizable model (+1 kcal mol^−1^) due to more attraction of the sodium ions and notable changes in peptide dynamics (see Supplementary Note 9).

## Discussion

We introduced the proposed polarizable model for gold and first examples for the simulation of interfaces and biomimetic processes on the 1 to 1000 nm scale. The validation across multiple metrics, including the lattice parameters of gold, the surface energy, the image potential, the polarizability, the hydration energy, peptide binding, and mechanical properties demonstrates very good reliability. Advantages over alternative models also include ease of use and realistic insights into Coulomb energy vs. van-der-Waals energy during adsorption and assembly processes. The model can capture the structure and dynamics of metal interfaces, in particular in the presence of ions, solvents, polymers, and biomacromolecules in atomic detail and is compatible with several force fields (AMBER, CHARMM, CVFF, GROMACS, IFF, OPLS-AA).

A large impact of induced charges is observed for ions in contact with metal surfaces in the gas phase, similar to the strength of covalent bonds (Figs 2 and 3). The contribution of induced charges to the adsorption of polar molecules such as water to metal surfaces in the gas phase is much smaller although it remains a notable contribution (Table 2). The polarizable model, as opposed to the nonpolarizable model^10^, can predict the differences. A large decrease in attractive polarization is observed from polar systems in the gas phase to polar systems in the condensed phase as a result of electrostatic screening (Fig. 4a, b). The magnitude of attractive polarization decreases more than an order of magnitude, which has important implications for the understanding of metal-aqueous, metal-oxide, metal-ceramic, and metal-polymer interfaces^67^. The contribution of polarization to adsorption amounts then to only to a fraction of a kcal mol^−1^ for polar molecules in contact with metal surfaces and up to a few kcal mol^−1^ per formula unit for the adhesion of ionic solids to metal surfaces. Contributions of polarization to interfacial contacts are therefore small compared to the energy of cohesion of metals, polar solids, and even liquids. As an example, the computed adsorption energies and density profiles of liquid water on gold (111) and (100) surfaces are almost identical using the polarizable and the earlier non-polarizable model (Table 2 and Fig. 5a)^10^. Excellent agreement with experimental data also underlines the importance to validate lattice parameters and surface energies of solids when developing force fields (Table 1)^9,68^. In comparison, computed surface energies of metals using DFT methods can incur 50% deviation relative to experiment^16^, which is more than ten times higher than with IFF and precludes meaningful predictions of interfacial properties.

Effects of polarization, even though on the order of intermolecular bonds, remain significant for the dynamics of ions and polyelectrolytes in contact with metal surfaces in solution. Polarization on the metal surface can temporarily open up the hydration shell of ions. Induced charges increase the attraction of sodium ions to the gold (111) surface, leading to a shorter distance and a larger negative adsorption energy (Fig. 5b). Larger chloride ion in solution, in contrast, showed a smaller increase in attraction due to polarization compared to sodium ions. Future studies with this model will be able to quantify specific interactions of metal surfaces with ions depending on the type of ion and the (h k l) metal surface. Polarizability also increases the adsorption energies of the FLAG peptide by ~10% due to changes in the dynamics of charged groups near gold (111) and (100) surfaces (Fig. 6). These findings are largely consistent with prior estimates by a-posteriori calculations of image potentials^26^. The new polarizable model indicates, however, that Na^+^_,_ NH_3_^+^, RCO_2_^−^ ions and similar ionic groups spend only a small percentage of time in direct contact with the (111) and (100) metal surfaces while polarizable atoms in the peptide backbone are strongly bound to the Au (111) surface via soft epitaxy. Contributions from electrostatic and van-der-Waals energy to adsorption can be identified, which is not feasible with a nonpolarizable model. The large negative binding energy of peptides to the gold (111) surface, for example, involves a positive contribution by Coulomb energy as the peptide is less polar than the replaced surface-bound water molecules, as well as a large negative contribution by van-der-Waals energy due to soft epitaxial interactions.

Most applications of metal nanostructures in living organisms, therapeutics, biosensors, electrode materials, batteries, catalysts, corrosion processes, and crystal growth^40,41,44,73^ are subject to some ionic strength, or externally applied potentials, or both, so that the use of a polarizable model is beneficial. In instances where solvents and solutes are neutral, the nonpolarizable model can be used^10^. The results reported here support the validity of earlier computational studies of ligands with no or few charged groups in contact with fcc metals in solution using the nonpolarizable model and the good agreement with experiment that has been demonstrated^5,13,17–22^. Nevertheless, the (hkl) surfaces, edges, and corners of metal nanocrystals are often covered by layers of chloride, bromide, or iodide ions from ionic surfactants (CTAB, CTAC) that induce substantial polarization effects with estimated contributions to binding up to 40%^21,64,69^. The growth and shape of nanostructures^70,71^, specific catalytic^30,40^, sensor^72^, and biological activity^2^ are affected by properties in the immediate vicinity of metal surfaces and benefit from modeling that captures precisely the relevant interactions^73^. Polarization interactions are also expected to be notable in mixtures of ionic and nonionic compounds in contact with metal surfaces. Even smaller effects on the order of few kcal mol^−1^ in ionic solutions can induce changes in protein folding and in the assembly of hierarchical superstructures containing DNA and lipid membranes.

The polarizable model can also be applied to understand surface and interfacial properties in the presence of external electric fields. However, it does not reproduce the internal electronic structure of the metal as the virtual electrons are tethered to the nuclei and not freely mobile (see Supplementary Notes 3 and 4). Therefore, the method of Siepmann and Sprik^34,35^ along with the prior non-polarizable potential^10^ can be employed to obtain true charge distributions within the metal for a given external potential. Additional assumptions for the charge distribution and on-the-fly calculation of variable electrode charges during molecular dynamics are then necessary. Alternatively, the polarizable model can be applied to simulate processes at electrodes at various potentials (Supplementary Fig. 7 and Supplementary Note 4). The initial distribution of net positive or negative surface charge must be physically chosen and polarization effects from the environment are then accounted for by the model without further adjustments. The advantage is the computation of reliable interfacial properties and reduced need for on-the-fly adjustments of the charge distribution. The true charge distribution inside the metal, if needed, can be revealed using the method of Siepmann and Sprik^34,35^. Follow-on studies will provide more details of the benefits and limitations of this model applied to electrode processes.

## Methods

### Interaction energy of single ions with the metal surface

The electrostatic attraction of ions to the metal surface was computed using energy minimization (molecular mechanics) (Figs 2b, 3e,f, Supplementary Figs 3–6). The Na^+^ or Cl^−^ ions were placed in an epitaxial position at a given distance from the metal surface atomic layer between 1 to 30 Å. The model systems were 3D periodic with a metal surface slab of lateral dimensions of 10 × 10 nm, 2 nm thickness, and 100 nm box height. These dimensions were required to ensure negligible influence of periodic images due to long range Coulomb interactions, and compute the image charge potential as a function of distance with <1% error (see Supplementary Table 2 and Supplementary Note 7). Standard Ewald summation, PPPM, and PME methods that are routinely available in molecular dynamics programs were employed to compute electrostatic interactions and include the image charge potential. The coordinates of the ion were fixed during energy minimization, or during NVT dynamics, to prevent movement towards the surface, which has no effect on the computed energy. Between one and three rounds of energy minimization of 200 steps each were carried out for each distance from the surface using the smart minimizer in Discover/Materials Studio^74^. The maximum derivative was small and convergence was reached when energies changed by less than 0.1 kcal mol^−1^. 200 steps energy minimization were typically sufficient, except for close distances with repulsive binding energies since then the metal surface slightly deforms in the vicinity of the ions. The final distance of the ion from the surface atomic plane, the total potential energy and all energy contributions (Coulomb energy, van-der-Waals (Lennard–Jones) energy, and bond energy) were recorded. Local deformation of the metal surface at close distance lead to slightly different final distances compared to initial distances related to the movement of the metal slab towards or away from the ions.

The energy profiles were complemented by energy minimizations without coordinate constraints of the ions, usually with start structures with ~3 Å initial distance. NVT molecular dynamics simulations were carried out with and without coordinate constraints on the ions at 298.15 K. All energy contributions including total energy, kinetic energy, Coulomb energy, van-der-Waals (Lennard–Jones) energy, and bond energy potential were recorded. The kinetic energy made essentially no contribution to ion adsorption at room temperature. Computed energy profiles, equilibrium distances, and adsorption energies from NVT molecular dynamics were the same as with energy minimization within 1% deviation. The cutoff for the summation of van-der-Waals (Lennard–Jones) interactions was 1.2 nm in all calculations. Coulomb interactions were computed using Ewald summation with high accuracy (10^−6^). The time step in molecular dynamics simulation was 1 fs. Longer (2 fs) and shorter time steps (0.5 fs) led to identical results. Temperature control was achieved by velocity scaling with a temperature window of ±10 K.

With regard to box size, the electrostatic interaction energy of Au (111) surfaces with Na^+^ and Cl^−^ ions in Fig. 2a was computed using an Au (111) surface with a thickness of 9 atomic layers (21.1 Å) and a box size of 100.93 × 99.90 × 1000.0 Å^3^. Total interaction energies and energy contributions in Fig. 2b and in Supplementary Fig. 3 were computed using models of Au (111) surfaces with 6 atomic layers and box dimensions of 100.93 × 99.90 × 140 Å^3^. Differences are not noticeable (see Supplementary Table 2).

Smaller model systems of Au (111) surfaces with a thickness of 6 atomic layers and 14.42 × 14.98 × 100 Å^3^ box dimensions were used to compare force field based calculations to DFT calculations in Fig. 3e, f and in Supplementary Fig. 6. These box dimensions represent the limit for quantum mechanical calculations. Finite-size effects are present, showing a slower decay of the total interaction energy with distance from the metal surface in the 10 to 20 Å range (Fig. 3e, f vs. Fig. 2b/Supplementary Fig. 3), as well as different electrostatic energies compared to data for the same system with 14.42 × 14.98 × 1000 Å^3^ box size (Fig. 3e, f vs. Supplementary Fig. 4, Supplementary Table 2, see details in Supplementary Note 7).

The analysis of the effect of partial polarization in Supplementary Fig. 5 was carried out on small Au (111) model systems with a thickness of 6 atomic layers and 20.2 × 20 × 84 Å^3^ box size. A given number of atomic layers on top of the surface included dummy electrons and the atomic layers underneath were represented by neat Lennard–Jones parameters without dummy electrons^10^. While the equilibrium interaction energy with Na^+^ ions was then −50 kcal mol^−1^ compared to −58 kcal mol^−1^ in large models (Fig. 2b and Supplementary Fig. 3), the effect of the number of polarizable layers on the polarization energy (the main result) is not altered by the finite system size.

The interaction energies can, alternatively, also be computed by steered molecular dynamics.

### Analytical calculation of the classical image potential

The classical image potential is a fundamental result from classical electrostatics and describes the interaction of point charges with a solid surface containing a free electron gas^26,75,76^. The classical image potential *E*_im_ in Fig. 2a and in Supplementary Fig. 4 was calculated as

1 $$E_{{\mathrm{im}}} = \frac{1}{{4\pi \varepsilon _0}}\frac{{ - q^2}}{{4(z - z_{{\mathrm{im}}})}},$$

where by *ε*_0_ is the dielectric constant in vacuum, *q* is the charge of the ion in units of the elementary charge *e*, *z* is the vertical coordinate of the ion, *z*_im_ is the vertical coordinate of the jellium edge above the metal surface atomic plane (or of the image plane more generally). The computed value of *E*_im_ from eq (1) is obtained in J. The image potentials in Fig. 2a and in Supplementary Fig. 4 are reported as a molar quantity in kcal mol^−1^, obtained upon multiplication of the result from eq (1) with Avogadro’s constant (*N*_A_ = 6.022137.10^23^ mol^−1^) and unit conversion from J to kcal mol^−1^.

Complex calculations of image potentials for a distribution of charges as described earlier^26^ (e.g., Equation 2.4 in ref^26^.) are no longer necessary with the new model. The image potential of a single charge was validated and the force field parameters were assigned (Fig. 1d) so that subsequently the virtual electrons on the metal atoms respond appropriately to any charge distribution brought into their vicinity and yield the image potential using a standard summation of electrostatic interactions without further modifications.

### Dependence of ion-gold interaction energy on box size

The interaction energies of the ions with the gold surface as a function of distance were evaluated for their dependence on the thickness and lateral dimensions of the gold slab, as well as the box height. The interaction energy with the gold surface increases up to a thickness of six atomic layers and remains effectively constant for any larger number of atomic layers (<1% increase). The convergence is related to the range of van-der-Waals interactions of about 1.2 nm (5–6 layers), and a slab thickness under 6 atomic layers is not suited to represent properties of bulk metallic surfaces.

Lateral dimensions of at least 7.5 × 7.5 nm^2^ and 20 nm box height are recommended in vacuum to keep finite size effects on image charge potentials below 1–2%. The computed image potential is of <1% error for dimensions larger than 10 × 10 × 100 nm^3^. Smaller lateral dimensions diminish the attraction of ions relative to infinite systems because the cloud of induced atomic dipoles can then not fully develop across the surface due to the proximity of neighbor cells.

At smaller lateral dimensions, a lower box height increases the attraction of ions to the polarized metal surface at short distance due to the spatial distribution of the charge in the cloud of induced dipoles near the metal surfaces, which increases the relative permittivity and diminishes repulsion between vertical periodic images of the ions. At larger distance of the ions from the surface, induced charges become negligible and repulsion between vertical periodic images of the ions is no longer reduced (see Supplementary Table 2 and details in Supplementary Note 7).

### Bulk and surface properties

The calculations of lattice parameters, surface energy, and mechanical properties followed earlier protocols using NPT molecular dynamics simulations^10^. Models of at least 20 × 20 × 20 Å^3^ size were employed in the computation of lattice parameters, density, and mechanical properties. The computation involved a series of NPT molecular dynamics simulations under applied triaxial stress for the bulk modulus, uniaxial stress for the Young’s modulus, and shear stress for the shear modulus. Typical applied stresses were between 0 and 2 GPa, corresponding to between 0% and 2% strain, and at least five data points were included for each modulus reported (Supplementary Table 1). Boxes of at least 20 × 20 × 120 Å^3^ size and NVT molecular dynamics simulations were employed in the computation of surface energies (cleavage energies), whereby 40 Å of the 120 Å height corresponds to the combined thickness of metal slabs and 80 Å to the combined thickness of vacuum slabs.

### DFT calculations of the attraction of single ions to the gold surface

The calculation of the attraction energies of single ions at the DFT level (Fig. 3e,f) was performed on the smaller model systems of Au (111) surfaces with six atomic layers and dimensions of 14.42 × 14.98 × 100 Å^3^. The same dimensions were used with the force field for an exact comparison. All calculations were carried out with the CP2K program package^77^ using the double-ζ valence plus polarization (DZVP) basis sets of the Molopt-type^78^ to represent the valence electrons. The interactions between valence and core electrons were described by the norm-conserving Goedecker, Teter, and Hutter pseudopotentials^79–81^. The Perdew–Burke–Ernzerhof (PBE) functional^82^ was used to model the exchange and correlation potential. Van der Waals interactions were accounted for by employing Grimme’s D3 dispersion correction^83^. The energy cutoff for the auxiliary plane wave expansion of the density was set to 400 Ry.

The attraction energy of the ions to the metal surface was computed using a series of geometry optimizations in which the Na^+^ or Cl^−^ ions were placed in an epitaxial position at several distances from the metal surface atomic layer in the range as described for the force field–based calculations. The coordinates of the ion were fixed during the geometry optimization to prevent movement towards the surface. For each distance from the surface, a convergence criterion of 3.0 10^−4^ hartree·bohr^−1^ was used for the maximum force component of the ionic configuration and a convergence criterion of 3.0 10^−7^ hartree was set as target accuracy for the SCF convergence.

At short range, the agreement between the force field–based and the DFT-based attraction energies is very good, however, DFT does not reproduce the physically expected 1/*r* convergence of the image charge potential to zero in the long range. This is a well-known problem of GGA density functionals (therefore also of the PBE functional) related to a strong electron delocalization error and an inaccurate description of the long range van der Waals interaction (see further references in Supplementary Note 6)^53,54^.

### DFT calculations of the difference electron density

Electron density differences Δ*ρ*_elec_ were calculated to investigate changes in the molecular electronic distribution induced by the metal across the metal-ion interface (Fig. 3a–d) and the metal-water interface (Fig. 4c). Δ*ρ*_elec_ was obtained as grid-based difference between the electronic density of the complex (slab + molecule(s)) ${\mathrm{\Delta }}\rho _{{\mathrm{elec}}}^{{\mathrm{slab}} + {\mathrm{mol}}}$ and the electronic densities of the individual components, keeping the atomic coordinates fixed to the positions they have in the complex:

2 $${\mathrm{\Delta }}\rho _{{\mathrm{elec}}} = {\mathrm{\Delta }}\rho _{{\mathrm{elec}}}^{{\mathrm{slab}} + {\mathrm{mol}}} + {\mathrm{\Delta }}\rho _{{\mathrm{elec}}}^{{\mathrm{slab}}} + {\mathrm{\Delta }}\rho _{{\mathrm{elec}}}^{{\mathrm{mol}}}.$$

For the purpose of representation, we also introduce a one-dimensional electron density difference integrated in the planes parallel to the surface (Fig. 4c), namely:

3 $${\mathrm{\Delta }}\rho _{{\mathrm{elec}}}^{{\mathrm{1D}}} = {\int} {{\mathrm{\Delta }}\rho _{{\mathrm{elec}}}\mathrm{d}x\mathrm{d}y}$$

### Computation of gold-water interfacial properties

The computation of adsorption energies of single water molecules, water monolayers, hydration energies, solid-liquid interfacial energies, and density profiles using the polarizable vs. the equivalent non-polarizable force field (Figs 4a, 5a, Table 1) was carried out using NVT and NPT molecular dynamics simulations. The protocols follow multi-box approaches (Supplementary Fig. 8)^84^.

Models of Au (111) surfaces of 28.84 × 29.97 Å^2^ area and 2 nm thickness, as well as models of Au (100) surfaces of 28.5474 × 28.5474 Å^2^ area and 2 nm thickness were employed. The adsorption energy of single water molecules was computed with three detached water molecules on and off the surface in the NVT ensemble, considering parts of the trajectory with at least 12 Å distance between all water molecules and their associated block averages of energies (Supplementary Fig. 8a). The simulation time was 3 ns with a time step of 1 fs, a spherical cutoff of 12 Å for van-der-Waals interactions, and Ewald summation of Coulomb interactions in high accuracy (10^−6^). The temperature was kept constant at 298.15 K. The first nanosecond was discarded for equilibration, and the last 2 ns employed to record block averages of thermodynamic properties. The average energy was adjusted to the exact temperature of 298.150 K using the heat capacity of the respective simulation box to eliminate errors due to small temperature differences.

The same protocol was applied to compute the adsorption energy per water molecule in surface-adsorbed water monolayers (Supplementary Fig. 8b). Water monolayers on Au (111) and Au (100) surfaces occupy about 8.8 and 8.3 Å surface area per molecule, respectively. These area requirements correspond to 98 water molecules on each model surface of the chosen size. The chosen box height was high (3000 Å) to accommodate the molecules adsorbed on each surface and desorbed with at least 30 Å distance from each other in the two-box approach (Supplementary Fig. 8b).

The computation of hydration energies and solid-water interfacial energies followed a three-box approach with combined dimensions of at least 20 × 20 × 60 Å^3^ (Supplementary Fig. 8c, d). NPT molecular dynamics simulations up to 5 ns were carried out following a protocol otherwise identical to the NVT protocol described above. The pressure was controlled by Parinello–Rahman barostat at atmospheric pressure and a time step of 0.5 fs was employed to keep pressure fluctuations small. Gold-liquid interfacial tensions were obtained from the gold-water interfacial energy and addition of an entropy correction of ~+0.06 J m^−2^ as previously described^10^.

All calculations were carried out with the polarizable force field, as well as with the nonpolarizable force field (pure Lennard–Jones parameters for gold)^10^. The flexible SPC water model (CVFF and IFF) was employed for water, and other models such as TIP3P performed nearly the same.

### Ion attraction to the gold surface in solution

Steered molecular dynamics simulations were carried out to determine the free energy profile of the interaction of sodium and chloride ions with the Au (111) surface using the polarizable and the nonpolarizable model (Fig. 5b and Supplementary Fig. 9). A water slab with thickness >3 nm and 704 water molecules were employed. The relaxation of ions and water in a new position occurs within ~0.1 ns so that 15 ns simulation time was chosen to sample the free energy profile in high resolution.

For each ion/surface combination, 15 ns NVT dynamics with 0.5 fs time step were carried out using LAMMPS and the “fix smd” command^85^. The nearest neighbor list was refreshed every time step. A constant pulling velocity of *v* = 1 Å ns^−1^ was applied to a fixed point in *z* direction and the point coupled to the ion with a spring constant of *k* = 20 kcal mol^−1^ Å^−2^ (Supplementary Fig. 9). To avoid movement of the Au slab, the bottom atomic layer of the gold slab (opposite to the exposed surface) was also constrained in the *z* direction with a spring constant *k* = 20 kcal mol^−1^ Å^−2^. The ion and the gold slab were mobile in lateral directions (*x* and *y*). The position of the ions and thermodynamic properties were recorded every 25 fs, resulting in 600000 data points for each curve.

The analysis involved visualization with the VMD program, calculation of the free energy (potential of mean force, PMF) as a function of the coordinate of the fixed point, and conversion into the free energy as a function of the coordinate of the ion. Savitzky-Golay smoothing was applied to convert the scatter plots with 600,000 data points into the curves shown in Fig. 5b, and the average energy between 12 and 15 Å distance was set as zero energy level for each curve. The calculations were repeated two times, using 0.5 fs and 1 fs time step, leading to consistent results. The accuracy is ±0.05 kcal mol^−1^.

### Analysis of peptide adsorption

The analysis of conformations and adsorption energies of the charged peptide Flg-Na_3_ on the metal (111) and (100) surfaces in aqueous solution was carried out using NPT molecular dynamics simulation with the polarizable force field, as well as with the non-polarizable force field for comparison (Fig. 6). At least five start conformations of the peptide DYKDDDDK were prepared at pH = 7, which equals a charge state NH_3_^+^-D(−)YK(+)D(−)D(−)D(−)D(−)K(+)-CO_2_^−^ · 3 Na^+^. The start conformations included helices, extended conformations, random coils, as well as selected structures obtained over the course of molecular dynamics simulations with specific salt-bridges. Models of Au (111) surfaces of 31.7207 × 34.9636 × 16.4820 Å^3^ and of Au (100) surfaces of 32.6256 × 32.6256 × 16.3128 Å^3^ were utilized for all simulations, along with 2000 molecules of pre-equilibrated water.

The models of surfaces, water, and peptides in specific conformations were used to prepare combined structures with the peptide away from the gold surface and on the gold surfaces (initially within less than 4 Å distance). A two-box approach was employed to analyze conformations away from the surface and conformations adsorbed on the surface, as well as adsorption energies and their additive contributions using multiple replicas and advanced sampling techniques (Supplementary Fig. 10)^84^. More than ten different replicas of the peptides on each surface and in solution were generated. After equilibration, the most favorable peptide conformations on the polarizable surface and on the non-polarizable surface were swapped and subjected to further molecular dynamics simulation to verify lowest energy conformations without doubt. The typical protocol was (1) build the initial configuration, (2) carry out 500 steps energy minimization to remove close atomic contacts between peptides and water, (3) perform 5 ns NPT molecular dynamics at room temperature, (4) then fix cell parameters and run 5 ns NVT dynamics at 600 K to overcome energy barriers, (5) relieve the constraints and complete 5 ns NPT dynamics at room temperature, (6) select further replicas from the annealing run at 600 K for equilibration at 298.15 K as needed. The analysis of average energies and conformations was performed during the last 4 ns of the trajectories in step (3) and (5), or on blocks of these trajectories with particular conformations of interest. All average energies were corrected to the reference temperature of 298.15 K using the heat capacity of the system (obtained as the change in average energy upon heating by 20 K divided by the temperature difference 20 K). Often but not always trajectory (5) was lower in average energy and lead to preferred structures. Initial models were prepared using Materials Studio and subsequently converted into.pdb/.psf format using conversion tools supplied with the INTERAFCE force field (IFF)^9^. Molecular dynamics simulations were carried out using the CHARMM-IFF force field and the NAMD program for simulations^86^.

The analysis involved visualization of peptide conformations and the dynamics in solution and on the surface, including salt bridges and binding residues. Adsorption energies and individual energy contributions were obtained from block averages of the trajectories. Subtle differences in conformations and energies were identified (see Supplementary Note 9). Similar protocols were also previously used to identify equilibrium structures and energies^8,13,19–22,87^.

### Data availability

The new model is fully described by the parameters listed in Fig. 1d. Sample force field files in CHARMM27-IFF and CVFF-IFF format, as well as models to build gold (111), (100), and (110) surfaces are available as Supplementary Data set 1. All data are also available from the authors.

## Electronic supplementary material

Supplementary Information

Description of Additional Supplementary Files

Supplementary Movie 1

Supplementary Movie 2

Supplementary Movie 3

Supplementary Movie 4

Supplementary Movie 5

Supplementary Movie 6

Supplementary Movie 7

Supplementary Movie 8

Supplementary Data
