# Supplementary material for: Insight into induced charges at metal surfaces and biointerfaces using a polarizable Lennard–Jones potential
Source: Nat Commun. 2018 Feb 19;9:716. doi: 10.1038/s41467-018-03137-8 (PMC5818522; doi:10.1038/s41467-018-03137-8)
Supplement: Supplementary file 1 — Supplementary Information [file 41467_2018_3137_MOESM1_ESM.pdf]

Supplementary Information

for

Instantaneous Insight into Induced Charges at Metal Surfaces and Biointerfaces Using a Polarizable Lennard-Jones Potential

by

Isidro Lorenzo Geada, Hadi Ramezani-Dakhel, Tariq Jamil, Marialore Sulpizi, Hendrik Heinz

Table of Contents

Supplementary Figures S2

Supplementary Tables S15

Supplementary Note 1: Capabilities and Limitations of Prior Models S17

Supplementary Note 2: Model Derivation, Validation, and Usage S22

Supplementary Note 3: Limitations of the Polarizable Model S29

Supplementary Note 4: Possible Extensions of the Polarizable Model S32

Supplementary Note 5: Calculation of Polarizability and Response to Applied Potentials S33

Supplementary Note 6: Gold-Ion Interfaces in Vacuum S39

Supplementary Note 7: Range of Induced Charges and Required Dimensions of Surface Models S41

Supplementary Note 8: Gold-Aqueous Interfaces S44

Supplementary Note 9: Binding of Charged Peptides in Aqueous Solution to Gold Surfaces S46

Supplementary References S51

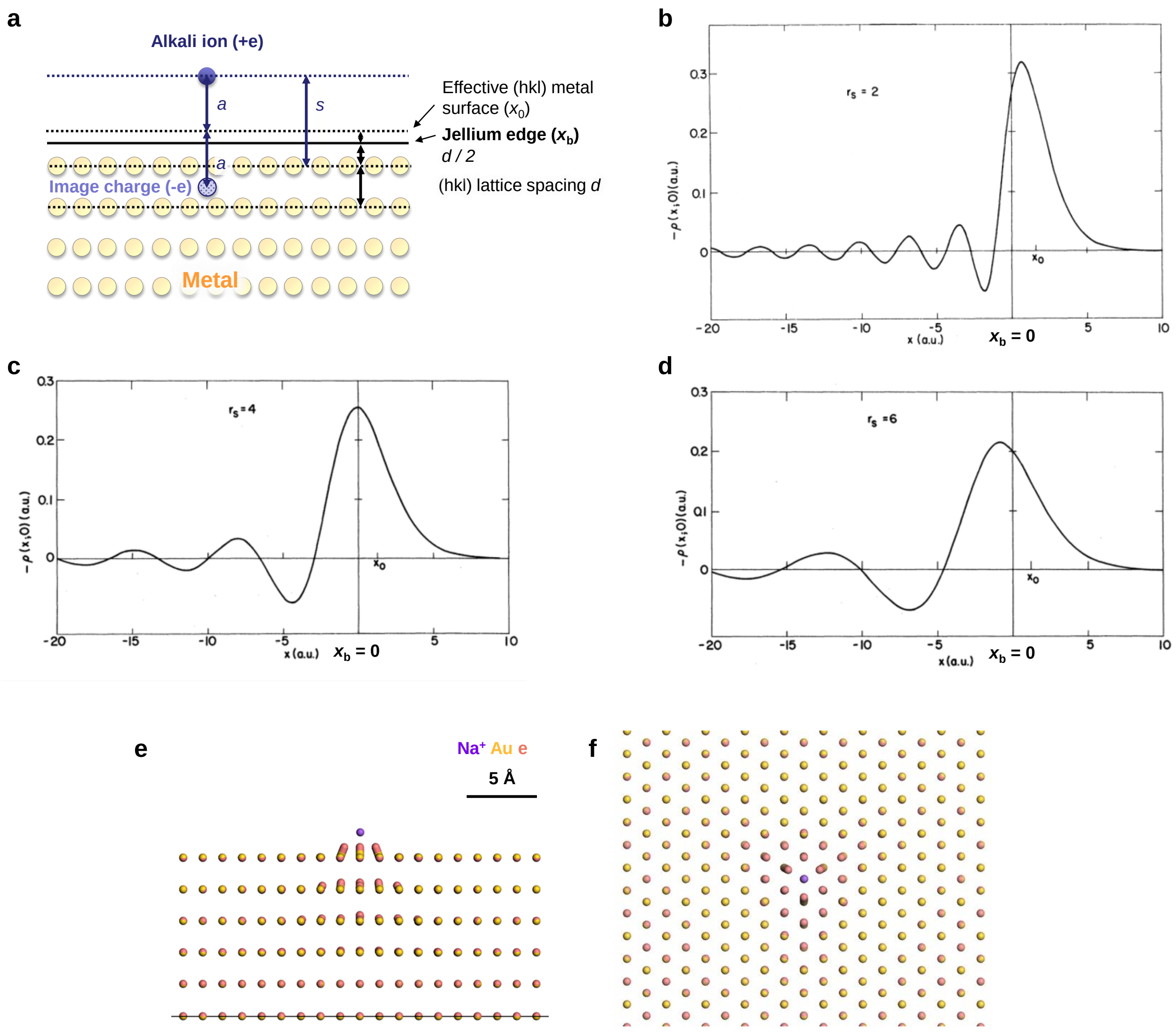

Supplementary Figure 1. Correlation of the proposed classical model with quantum mechanical findings. (a) The attraction of an alkali ion to a metal surface equals the interaction energy with an equidistant imaginary image charge on the other side of the effective metal surface. The effective (h k l) metal surface x_0 is located near the jellium edge x_b, which is a plane located half a lattice spacing *d* above the top layer of atoms (ref. [1]). *a* is the distance of the ion from the effective metal surface and *s* is its distance from the surface atomic plane. x_0 can be located on the either side of the jellium edge x_b (ref. [2]). The lattice spacing *d* perpendicular to a chosen (h k l) surface plane is 2.35 Å for gold (111) and similar to 2 Å for various other metals. (b-d) Friedel oscillations of the

induced electron density in the metal in the presence of an external alkali metal ion, which represent the quantum mechanically derived image potential. The units are 1 a. u. = 0.529 Å. The true induced charge is located near the metal surface and approaches zero at a distance of ~10 Å inside the metal surface, corresponding to about five atomic layers. Different values of r_s characterize different bulk electron densities. Reproduced with permission from Lang and Kohn, ref. [1]. (e) Snapshots from energy minimization using the polarizable force field show the displacement of dummy electrons from the atomic centers, which subsides at about four to five atomic layers below the surface (side view). (f) Top view onto the sodium ion in contact with the metal surface using the polarizable force field. The image charges also propagate several atomic layers laterally. The response in the classical polarizable model agrees in length and depth with the dimensions of quantum mechanical Friedel fluctuations. The data are shown for the Au (111) surface, and other (h k l) surfaces lead to similar results.

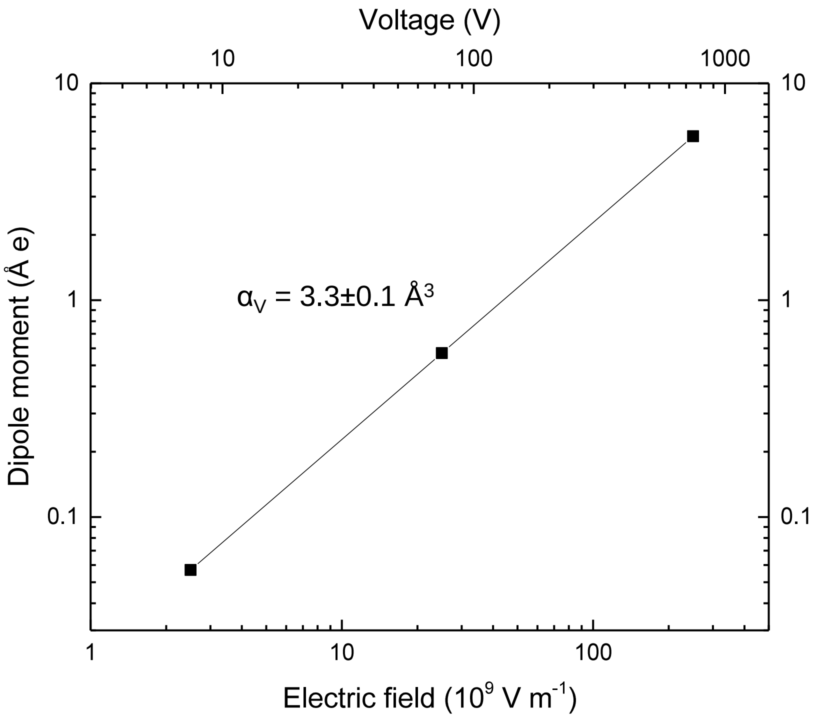

Supplementary Figure 2. Response of a single Au atom between two charged infinite parallel plates to an applied electric field (and equivalent voltage) using simulations with the classical polarizable model. The induced dipole moment μ increases linearly with the applied electric field. The polarizability α_V corresponds to the slope of $\mu = f(E) = \alpha \cdot E$ (normalized by $4\pi\varepsilon_0$) and matches the analytical result $\alpha_V = \dfrac{q^2}{8\pi\varepsilon_0 k_r}$, both yielding α_V = 3.3±0.1 Å^3.

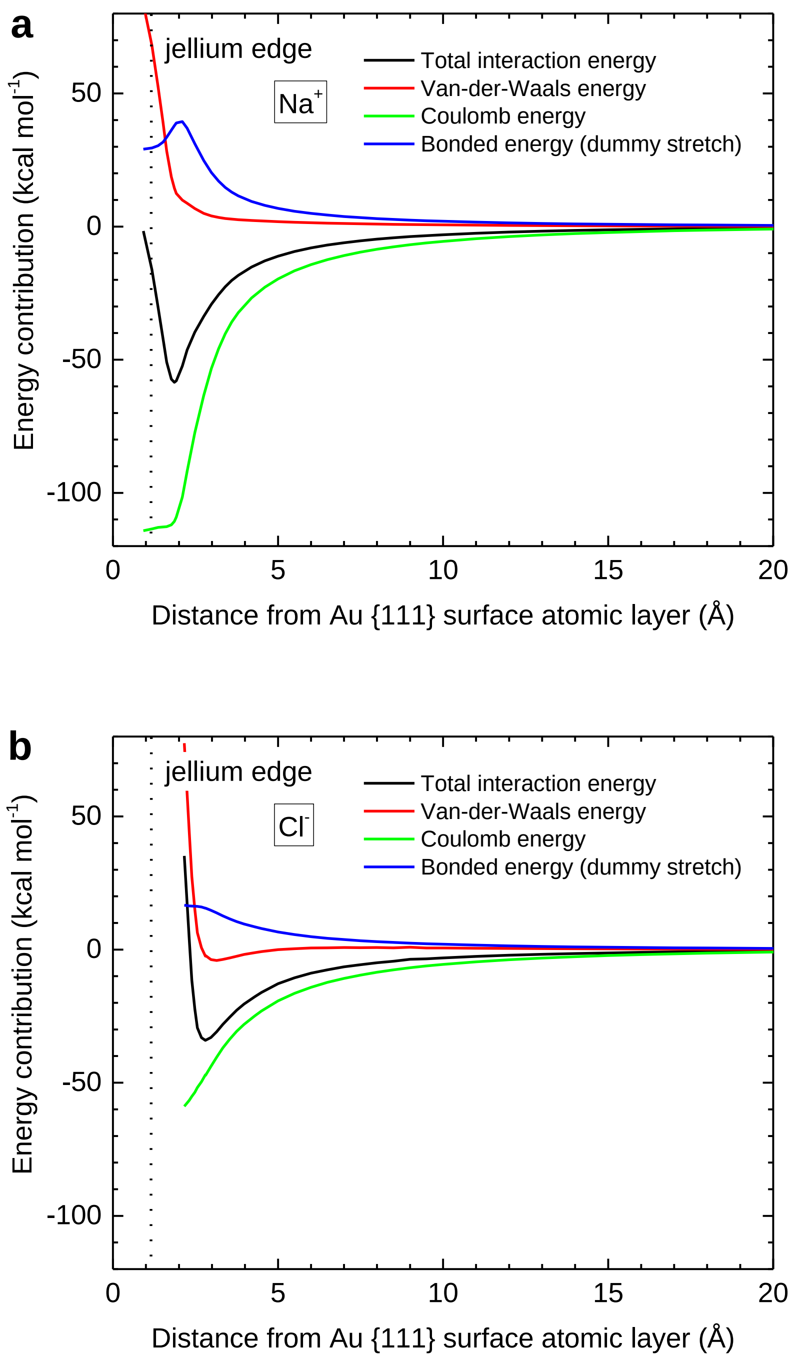

Supplementary Figure 3. Breakdown of energy contributions for the adsorption of Na^+ and Cl^- ions in vacuum to the Au (111) surface using the polarizable force field. A large surface area of 10×10 nm^2 and a height of 14 nm of the periodic simulation box were used to eliminate finite-size

effects. (a) Na^+ ion. (b) Cl^- ion. The attractive Coulomb energy decreases to larger negative values as the ions approach the surface (green curve). In response, dummy electrons are pulled outward from the gold atom cores in case of Na^+ ions, or pushed inwards in case of Cl^- ions, leading to a positive bond stretching energy (blue curve). The bond stretching energy increases the total energy of the system and partly offsets the negative Coulomb energy at short distances. It is internal to the metal and does not diminish the attraction of adsorbed species. The repulsive contribution of the van-der-Waals energy (red curve) becomes significant at close distance and keeps the ions at equilibrium distance away from the surface. The total energy is the sum of these contributions (black curve).

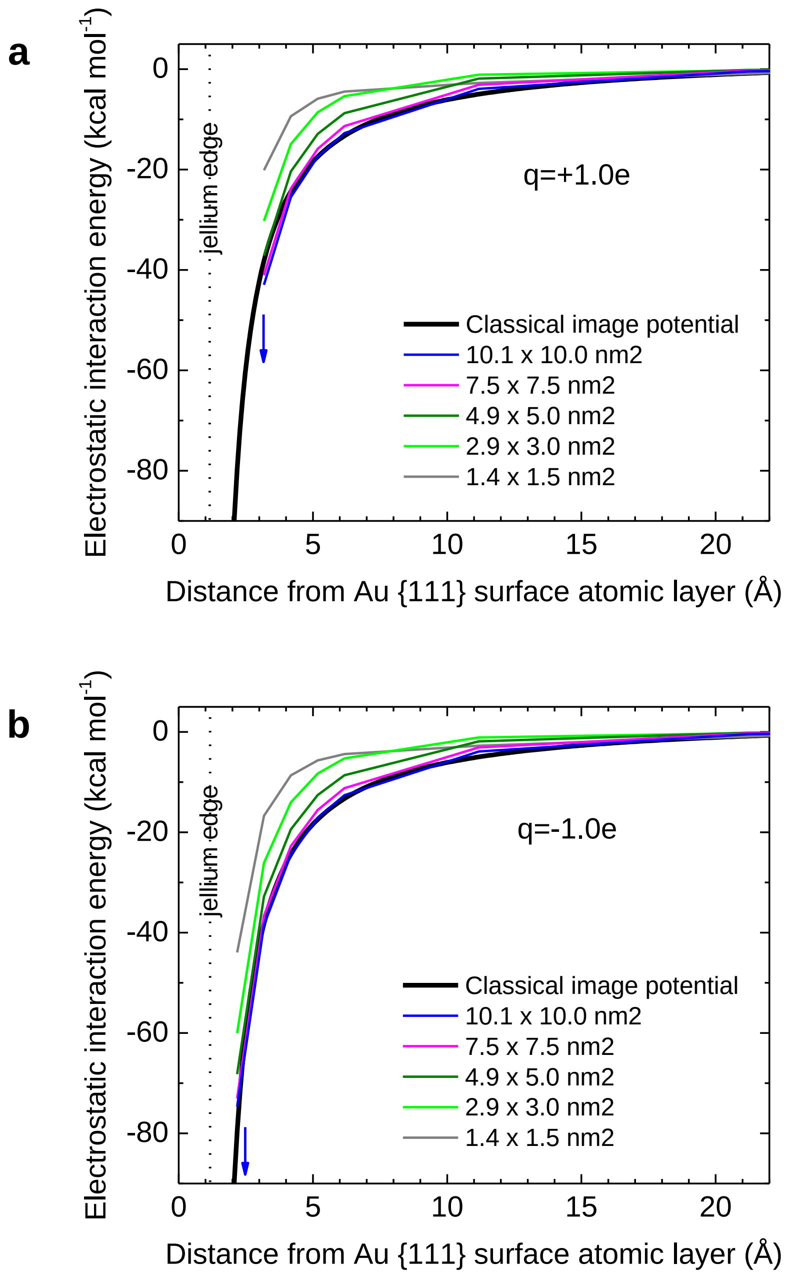

Supplementary Figure 4. Electrostatic interaction energy for (a) a positive and (b) a negative point charge with the Au (111) surface in vacuum according to the polarizable model for different horizontal dimensions of the 3D periodic simulation box and 100 nm box height, showing the dependence on lateral dimensions. The classical image potential corresponds to the limit of infinite dimensions and is reproduced in excellent agreement by the force field for side lengths of >7.5 nm

(black curve). For smaller lateral box dimensions, the induced surface dipoles cannot fully develop over several atomic layers due to interference with periodic neighbor cells, and attractive polarization is reduced. The response is nearly identical for positive and negative charges. The image potential is only meaningful to describe attraction for distances from the jellium edge (image plane) between ~2.5 Å to $+\infty$ (refs. [3], [4]).

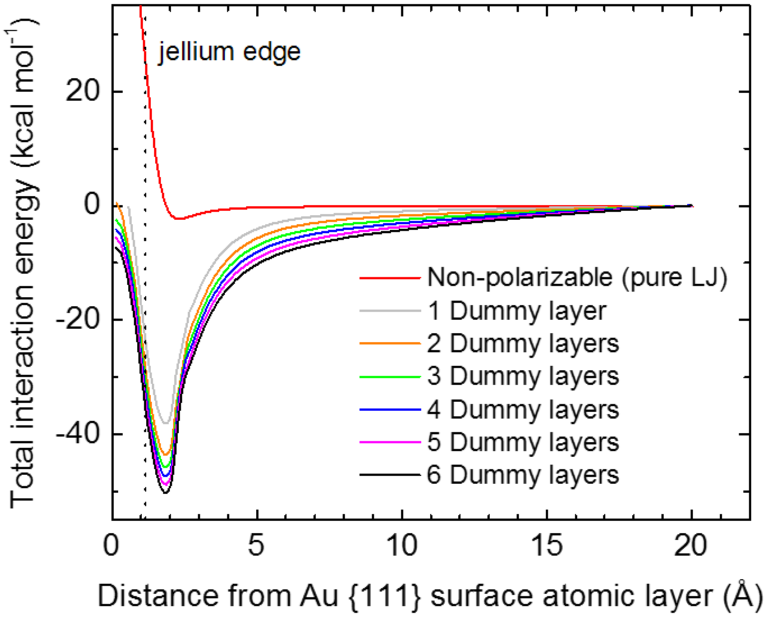

Supplementary Figure 5. Total interaction energy of a Na^+ ion with the gold (111) surface as a function of the number of atomic layers on the gold surface modified with polarizable dummy electrons. One layer with dummy electrons accounts for 70% of the total polarization energy. Best accuracy is reached when several layers or all atoms include dummy electrons. The box size was 2.0×2.0×8.4 nm^3.

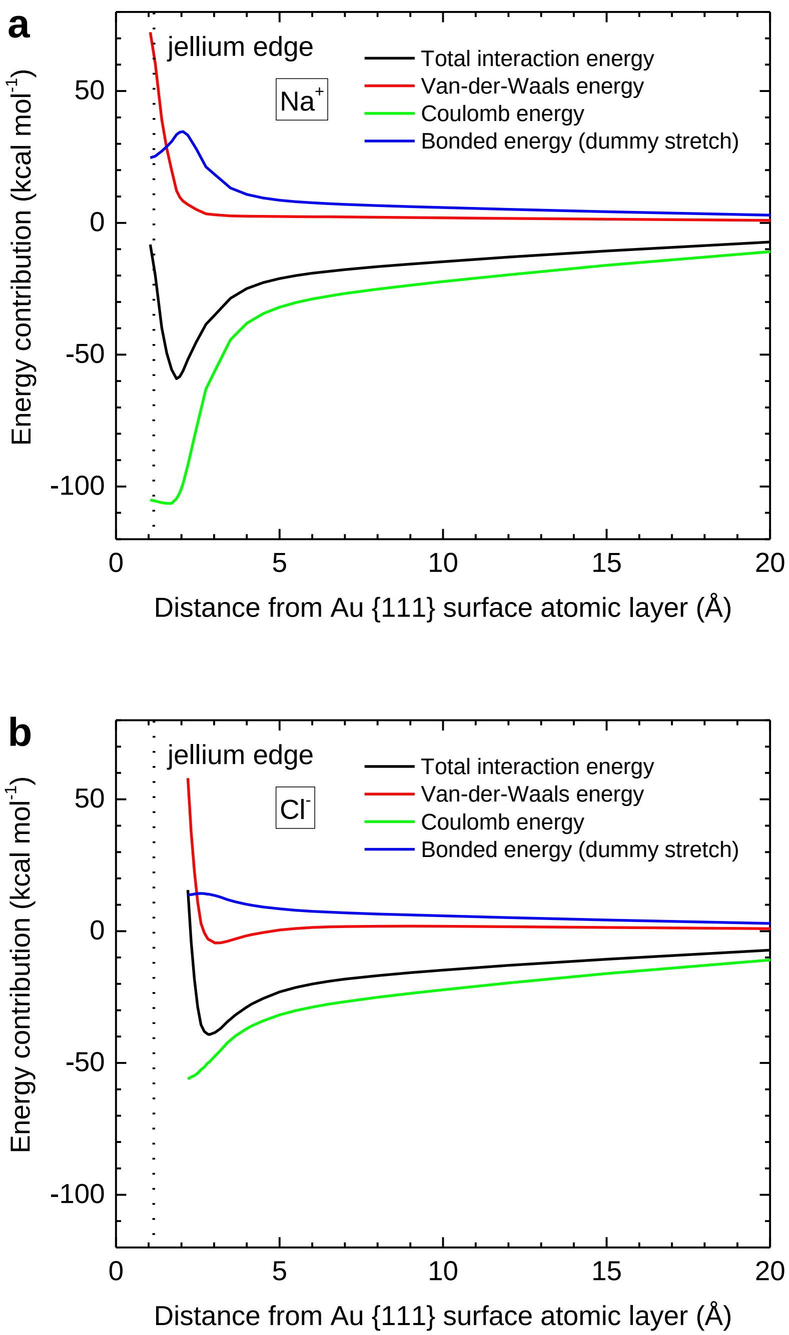

Supplementary Figure 6. Breakdown of energy contributions for the adsorption of Na^+ and Cl^- ions in vacuum to the Au (111) surface with a small surface area using the polarizable force field, only meaningful for comparison with DFT calculations (Fig. 3). (a) Na^+ ion. (b) Cl^- ion. The box

dimensions are 1.44×1.50×10 nm^3 and data for a larger simulation box without finite size effects are shown in Supplementary Fig. 3.

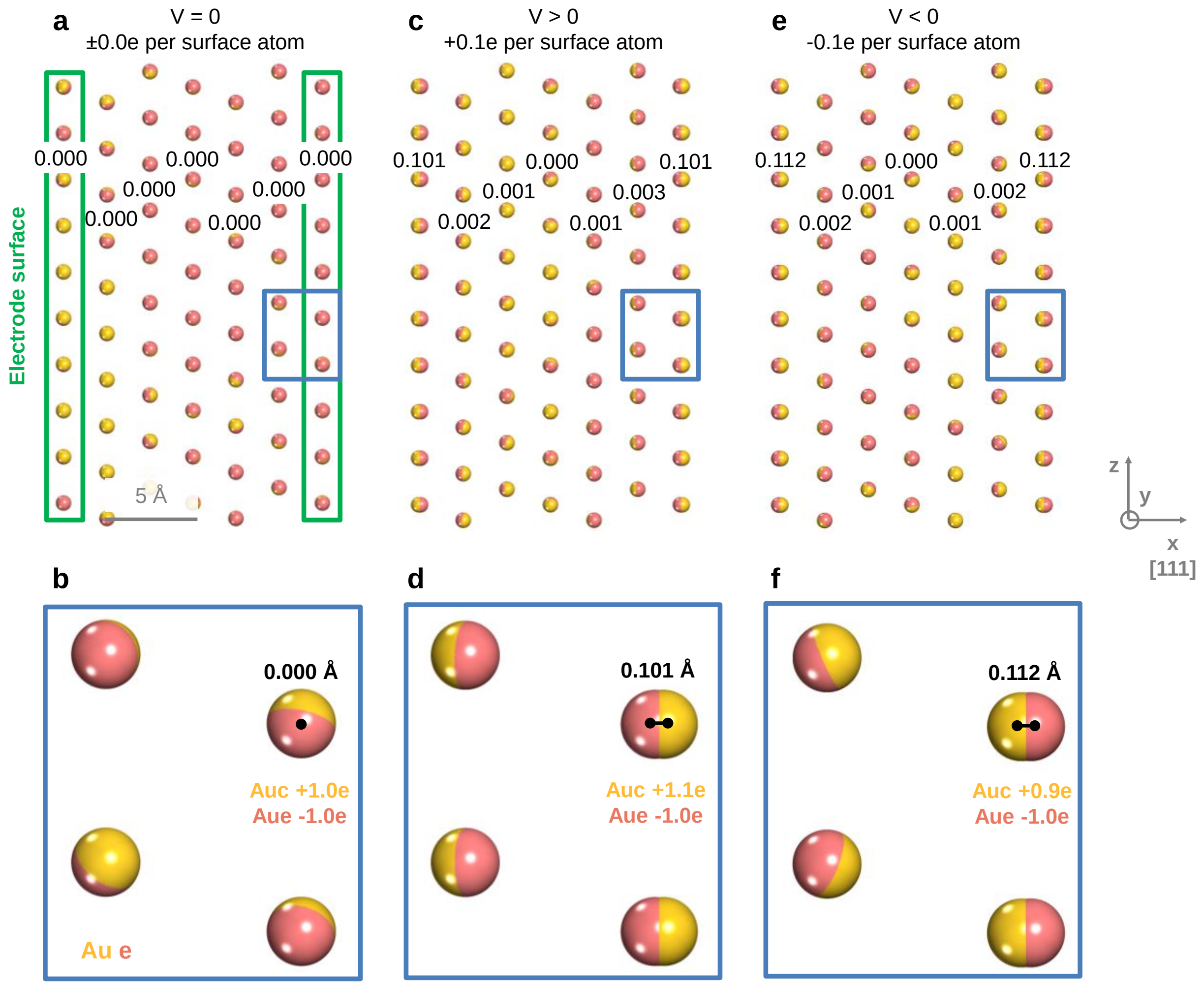

Supplementary Figure 7. Response of the polarizable model for gold to an applied electrical potential. A thin metal slab represents an electrode with two (111) surfaces perpendicular to the *x* direction and periodicity in the *y* and *z* directions (seven atomic layers × 2.31 nm × 2.50 nm). The electrode surfaces carry net charges that are distributed evenly onto the nuclei of the top atomic layer. (a, b) The model without an applied voltage. The net charge on the electrode surface is zero

and all dummy electrons rest at the atomic centers. The numbers in black color represent the displacement of the dummy electrons in Å in each atomic layer after energy minimization, proportional to the strength of the local electric field. (c, d) The model with a positive voltage applied. The voltage is represented by a discrete distribution of charges equivalent to +0.1e per surface atom (d). The net positive charge on the outer surface atoms leads to an inward orientation of the virtual electrons by ~0.1 Å (d) and the inner portion of the metal slab remains field free as seen by the negligible displacement of dummy electrons in (c). (e, f) The model with a negative voltage applied. The voltage is represented by a discrete distribution of charges equivalent to -0.1e per surface atom (f). The net negative charge on the outer surface atoms leads to an outward orientation of the virtual electrons by ~0.1 Å (f) and the inner portion of the metal slab remains field free (e). Removal of the voltage leads reversibly back to state (a), and the applied voltages on the metal surface do not affect the interior of the metal. After a voltage is applied, the model continues to reproduce the magnitude of polarization by an external charge distribution in the environment (ions and molecules) without on-the-fly adjustments. As a limitation, the internal electronic structure of the metal is not realistic due to lack of full charge mobility. The initial distribution of net charges on the metal surface and within the metal should be physically informed and can be updated periodically. Details remain to be explored in follow-on studies.

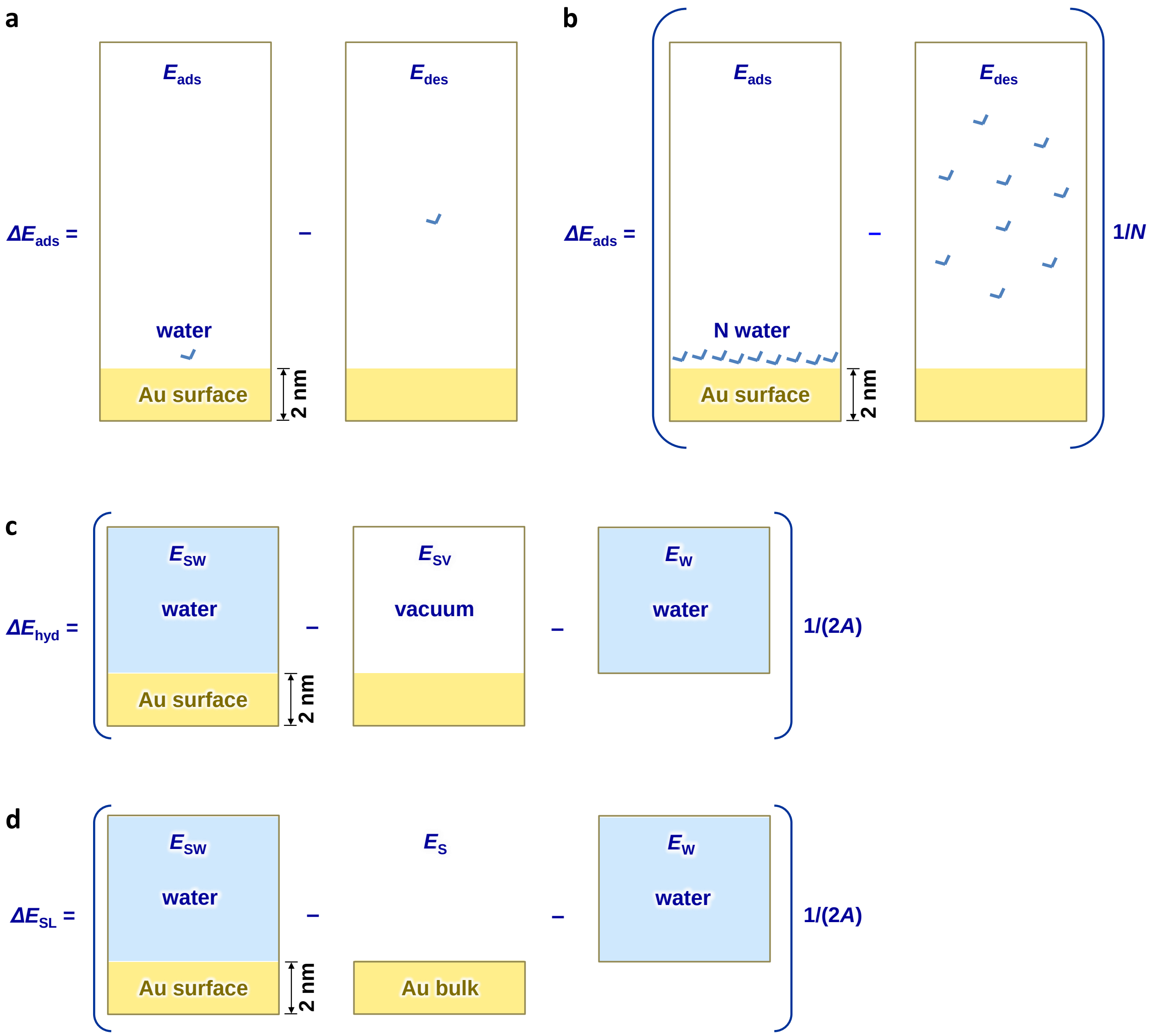

Supplementary Figure 8. Schematic of the computation of the adsorption energy, hydration energy, and solid-water interfacial energy. (a) Adsorption energy of a single water molecule. (b) Adsorption energy of water in a molecular monolayer that consists of *N* water molecules on a gold surface, normalized per water molecule. The adsorption energy is the difference in average equilibrium energies in the adsorbed state *versus* the in the desorbed state. The box height is chosen high enough such that water molecules in the desorbed state do not interact with each other nor with the Au surface (initial distances greater than 3.0 nm to avoid approaches closer than 1.2 nm during molecular dynamics). The height of the simulation boxes with 98 water molecules was

therefore about 300 nm (not drawn to scale). (c) Computation of the hydration energy involves three simulation boxes and the energy is normalized by the interfacial area 2*A*. (d) Computation of the metal-water interfacial energy normalized by interfacial area 2*A*. Average energies for all simulation boxes were obtained from NPT molecular dynamics simulations.

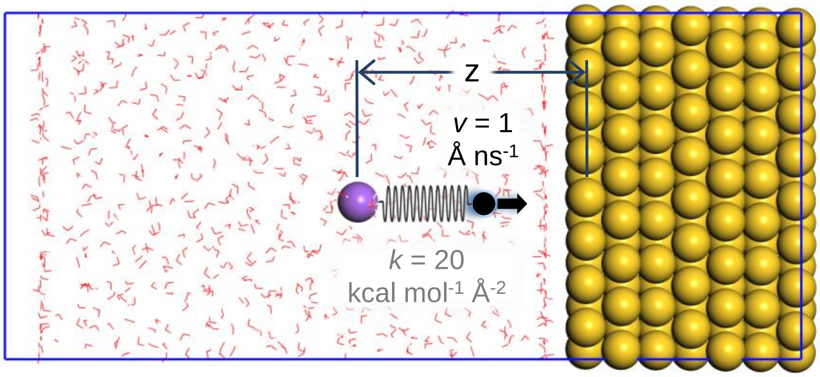

Supplementary Figure 9. Illustration of steered molecular dynamics to obtain the free energy profile (potential of mean force) for sodium and chloride ions as a function of distance from the metal surface.

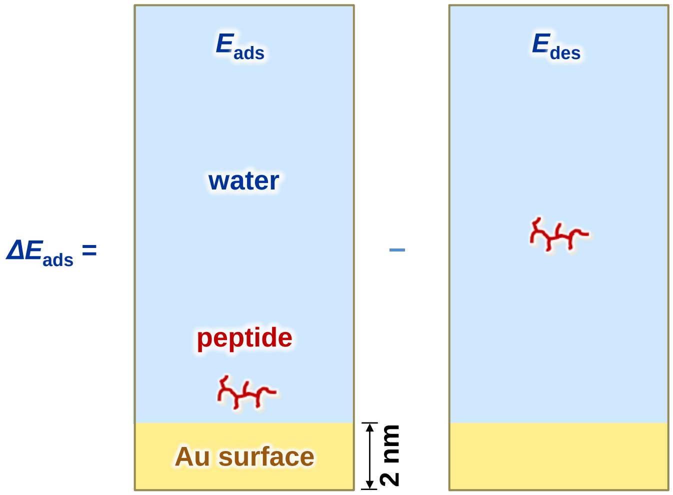

Supplementary Figure 10. Schematic of the computation of the adsorption energy of the peptide Flg-Na_3 on gold surfaces. The difference in average equilibrium energy is computed for the two boxes with the peptide in adsorbed and desorbed states, respectively. Conformation sampling using independent replicas was essential to identify conformations of minimum energy on the respective (h k l) surfaces and in solution.

Supplementary Table 1. Isotropic elastic constants E (in GPa), K (in GPa), G (in GPa), and ν in experiment and in the simulation with the polarizable model (Aue) and with the nonpolarizable model (Au).

Model	Young's Modulus E	Bulk Modulus K	Shear Modulus G	Poisson Ratio ν
Expt[a]	88±3	173±6	31.1±1	0.415±0.005
Aue	105	145	40	0.38
Au[b]	110	133	41	0.36

[a] Refs. [5, 6]. Experimental results differ between independent sources up to a few percent at 298 K.

[b] Ref. [7].

Supplementary Table 2. Dependence of the Coulomb contribution of the equilibrium adsorption energy of a Cl^- ion to the gold surface in vacuum E_{Coul} on the lateral dimensions and on the height of the simulation box (at 2.82 Å distance relative to 30 Å distance). Rows highlighted in green indicate convergence to continuum data and a minimum size of roughly 80×80×1000 $Å^3$ for errors <1%. Coulomb energies were computed in high accuracy (10^{-6}).

Lateral size ($Å^2$)	Height (Å)	E_{Coul} (kcal mol^{-1})
14.4×15	10000	+15.6
72×75	10000	-45.1
14.4×15	1000	-19.2
72×75	1000	-44.7
14.4×15	100	-56.3
72×75	100	-46.1

Supplementary Note 1: Capabilities and Limitations of Prior Models

This note describes prior models for polarization of metals and charge transport, the relationship to the proposed model, and the challenges solved. Earlier polarizable models have been associated with the groundbreaking treatises on electron theory in metals by Paul Drude[8, 9] and on the motion of electrons in metallic bodies by Hendrik Lorentz.[10, 11, 12] Drude assumed delocalization of the electrons[8] and Lorentz proposed a more classically inspired potential of attachment of electrons to nuclei[10] in the discussion of electric effects. The nature of protons and electrons was still unclear at the time, for example, Drude left it open whether nuclei or electrons could diffuse, and the connection to current day energy functions in computer models is therefore only conceptual.

Core-shell models, or charge-on-spring models, were first developed for ionic salts[13, 14, 15] and later extended to liquids and biomolecules.[16, 17] For more recent models that include explicit charged particles attached to an atom via a harmonic spring, the term "Drude oscillator models" has been coined.[16, 17] These charge-on-spring models are very similar to earlier core-shell models[15] and the term "Drude oscillator models" is a misnomer as Lorentz (p. 451)[10] rather than Drude[8] proposed classical potentials for electron-nucleus interactions. In the original work by Drude, the electrons have unrestricted motion as opposed to being flexibly attached to a nucleus, which occurs in today's "Drude oscillator models", and the original models by both Drude and Lorentz do not include long-range electrostatic interactions between the charged particles. The model for metals proposed here includes full range electrostatic interactions as well as a Lennard-Jones potential among all non-bonded particles (nuclei and electrons). These interactions are essential for the function of the model and have not been part of the original models by Drude, Lorentz, or earlier core-shell models including the so-called "Drude oscillator models".[13, 14, 15, 17] The proposed

polarizable model therefore uses a new energy function and is yet fully compatible with common energy expressions and bonded force fields.

Prior Core-Shell Models for Salts and Liquids. Core-shell models of ions in salts with a core charge and a shell charge of opposite sign have been developed several decades ago to qualitatively represent environment-dependent fluctuations in charge states.[13, 14, 15, 18, 19, 20] The models involve a net charge on every atom and Buckingham potentials that act on the ionic cores while the shells are excluded from Buckingham interactions. The models have been helpful to understand the polarization of ionic solids in electric fields. In contrast to metals, the charge densities of ions in salts are more localized and less variable depending on the chemical environment.[21, 22] In comparison, the proposed model for the metals involves a net charge of zero per atom and a Lennard-Jones potential that acts on the nuclei as well as on the virtual electrons.

Models that contain fixed off-site electrons for electron lone pairs in molecules have greatly improved property predictions by an order of magnitude. For example, the TIP5P water model includes the oxygen and hydrogen atoms, as well as two dummy electrons that represent the sp^3 hybridized lone pairs of oxygen.[23] In contrast to the proposed model for metals, however, the TIP5P model is not polarizable.

Polarizable models with flexible charge-on-spring models have been developed for liquids and biomacromolecules.[15, 17] Harmonic springs are used for added charges as in the proposed polarizable model for gold. However, a Lennard-Jones potential is not applied to interactions between all non-bonded charged sites, and often additional rules and empirical screening functions are added that are not necessary in the present model.

Modelling of Metallic Electrodes with Explicit Charge Distributions under Applied Electric Fields. Extensive efforts have been made in modelling of metallic electrodes and charge

distributions under applied electric fields.[2, 24, 25, 26, 27, 28, 29, 30, 31] Siepmann and Sprik[24] introduced models that use a variable Gaussian charge distribution to represent a constant potential at an electrode, and the charge distributions are adjusted on-the-fly according to a variational procedure. A great benefit of the Siepmann-Sprik model is that image potentials are reproduced (matched) using the chosen distribution of charges. The approach has been very valuable for the simulation of charged electrodes, especially in combination with ionic liquids. For example, the method provided first insights into metal-water interfaces at constant potential, albeit with some uncertainty.[24] A further development of the Siepmann-Sprik method by Reed et al. allows the simulation of interfaces of ionic liquids with metal surfaces under application of an external electrical potential.[25] The protocols by Siepmann and Sprik (1995)[24] have also been applied to study interfaces of electrodes with water and ions such as electrolyte interfaces[26] and Pt-water interfaces.[27] Among the results, the Poisson potential profile across pure water in a parallel plate capacitor was found to be in good agreement with the one expected from the dielectric constant and the classical analytical result.[26] This model has proven as a reliable tool. Downsides are that the validation of the surface energy of the metals and wetting properties in contact with solvents are not reported, and that explicit calculations of the charge distribution must be added to the molecular dynamics algorithm. The original energy functions were complex and needed to be customized for every compound interacting with the metal. The proposed polarizable model overcomes these limitations.

A method to compute induced charges (ICC) was also presented by Tyagi et al.[31] The method is, however, not applicable to a specific system as system-specific force field developments and validation are necessary. Merlet, Madden, and Salanne et al. carried out simulations of nanoporous carbon electrodes in contact with ionic liquids related to applications in supercapacitors.[28] The

charge distribution upon an applied electric field was solved using the method of Reed et al. (2007).[25] Simulations of ionic liquids in contact with three-layer graphene under an applied external field were also reported by Breitsprecher et al.[30] Onofrio and Strachan implemented charge distributions in reactive molecular dynamics of electrochemical processes under applied electric fields.[29] The inclusion of additional charge is feasible, however, the protocol neglects image potentials and is numerically complex. Validation of surface energies and hydration energies has not been reported.

Inclusion of Image Potentials without Explicit Charge Distributions. First models for on-the-fly inclusion of image charge potentials in metals in molecular dynamics simulations were introduced by Iori et al.[32] and have been applied to various gold interfaces with water, peptides, and organic ligands.[33, 34, 35, 36] In the original model,[32] metal atoms were fixed and rigid rod dipoles included to enable polarization. Difficulties arise as the response to positive *versus* negative charges adsorbed on the surface is asymmetric and the energy function was not evaluated in comparison to classical image potentials. Pensado et al. combined the rigid-rod potentials[32] with other potentials for metals to enable atom mobility.[37] Nevertheless, the validation of bulk properties, surface energies, interfacial properties, and image potentials remains limited. This group of polarizable potentials is also difficult to include in major force fields for materials and biomolecules such as AMBER, CHARMM (except for special cases with numerous added parameters[33]), CVFF, DREIDING, GROMACS/GROMOS, IFF, and OPLS-AA, restricting broad application.

In earlier work, we described the location of image charge planes and the possibility to quantitatively compute the image potential on metal surfaces in contact with condensed phases, including available experimental data on the location of image planes,[3] examples of water and

peptide interfaces, and a sensitivity analysis.[2, 7] While surface and interfacial properties of the metals match experimental data, calculations of image potentials were carried out a-posteriori and uncoupled from the dynamics. The resulting contributions from polarization including total energies and the decomposition into Coulomb and van-der-Waals interactions were therefore approximate. The a-posteriori calculations also require time-consuming extra coding and post-processing for flat surfaces, and cannot be applied to corrugated surfaces, or under applied electric fields.[2] The lessons learned from this prior study have contributed to the proposed model to overcome the limitations.

Challenges Solved by the New Model. The proposed classical polarizable model is simple, accurate, and compatible. It can be used for molecular simulations of metals and their materials and biological interfaces using several existing force fields (AMBER, CHARMM, CVFF, DREIDING, GROMOS/GROMACS, IFF, OPLS-AA) and standard molecular dynamics codes (Discover, Forcite, LAMMPS, NAMD, GROMACS) without further add-ins or modifications required. External potentials can be applied to understand resulting surface polarization and interfacial properties (see Supplementary Note 4).

The new model computes accurate polarization properties, bulk properties, and interfacial properties, which previous models alone cannot provide, and the implementation in existing force fields and molecular dynamics codes is straightforward. The model exceeds the accuracy of DFT calculations for surface energies and aqueous interfaces (if chemical reactions excluded), requires no assumptions for charge distributions, no add-in code during molecular dynamics, and no additional parameters beyond those listed in Fig. 1d. It retains all benefits of the non-polarizable model,[7] adding instantaneous polarization, responses to external potentials, and improvements in mechanical properties.

Supplementary Note 2: Model Derivation, Validation, and Usage

The main aim of the polarizable model has been the expansion of the well-performing Lennard-Jones parameters for fcc metals[7] to mimic features of the free electron gas. Specifically, we aimed at reproducing the classical image potential on-the-fly and, thereby, electrical polarization by external charges and potentials in molecular dynamics simulations. The properties of the free electron gas play an essential role as they are the physical cause for the image charge potential and for polarization of metal electrodes in electric fields. It is not possible, however, to translate quantum mechanical properties of free electrons into a much simpler and faster classical model without accepting several approximations. Our focus has been on accuracy and wide applicability of the new polarizable potential, including the extension to other metals. Therefore, we focused on the physically most necessary, simple, and practical additions to the existing particle-based Lennard-Jones models.[7] According to this motivation, details of the derivation, validation, and usage of the resulting polarizable model are described. Limitations and possible extensions are discussed in Supplementary Notes 3 and 4.

Derivation. Mobile dummy electrons with a charge on the order of -1*e* were added to the atomic centers that carry a compensating positive charge on the order of +1*e*, whereby the nucleus and the virtual electron are coupled via a harmonic spring. This choice avoids the challenge to represent a truly “free” electron gas in a classical model, which would require further parameters to describe the motion of virtual electrons and suffer from a particle-based representation. The charge q and the bond stretch constant k_r were tuned to reproduce the classical image potential, which is determined by the polarizability $\alpha = q^2/(2k_r)$ and vice versa (see Supplementary Note 5 for details). The polarizable model has five independent parameters including the mass of the dummy electron, q^2/k_r, σ, and two ε values (Fig. 1).

The standard exclusion of nonbond interactions between 1, 2 bonded atoms in classical force fields is hereby vital to the function of the model. The exclusion eliminates Coulomb and van-der-Waals interactions between core-electron pairs of the same atom.

An equilibrium displacement of zero was chosen for the dummy electron ($r_0 = 0$ Å), an appropriate bond stretching constant k_r, as well as Lennard-Jones parameters for atom cores and dummy electrons (Fig. 1). The mass of the dummy electron was chosen to be small in comparison to the metal (<1%) so that it can be considered as a perturbation with a near-negligible effect on the dynamics of the atomic center (1 a. u. *versus* 197 a. u. for gold-plus-dummy). This ratio of about 0.5% can also be considered a fixed value, especially when considering extensions to other metals, and then reducing the total number of independent parameters to four. The negatively charged dummy electron behaves similar to a hydrogen atom and displaces up to about 0.5 Å from the atomic center in the presence of a charged ion (±1*e* and ±2*e*). The displacement remains well below 0.8 Å to avoid failure modes of approaching the jellium edge at >1.0 Å and recombination with cations or neighbor atom cores. To prevent such failure modes and guarantee stability up to high temperature, Lennard-Jones parameters σ and ε were introduced for the dummy electrons, not only for the atom cores. The purpose of the LJ parameters on the dummy electron is endowment with repulsion to prevent close approaches of external ions and neighbor atom cores. The nonbond diameter σ_e of the dummy electron was set equal to σ_{core} of the atom core. This assignment reinforces an equilibrium position of the dummy electron at the atomic center, leading to an energy penalty from bond stretching and from repulsive LJ interactions with the nonbonded neighbor atoms upon elongation. The condition $\sigma = \sigma_{core} = \sigma_e$ also helps prevent spontaneous polarization in molecular dynamics and keeps the number of adjustable parameters low. The value of σ for gold in the polarizable model (2.946 Å) is almost the same as the value of σ in the non-polarizable

model (2.951 Å) (Fig. 1d). The ε parameters for the atom core and the dummy electrons in the polarizable model (3.50 and 0.20 kcal mol^{-1}) are smaller than the ε parameter for gold in the non-polarizable model (5.32 kcal mol^{-1}),[7] respectively.

An aspect worth noting hereby is that the polarizable model qualitatively relies on the Born-Oppenheimer approximation, which assumes that the motion of atomic nuclei and electrons in a molecule can be separated.[38] Dummy electrons have only 0.5% of the mass of gold and move significantly faster than the metal nuclei. At first sight, the Born-Oppenheimer approximation for the "coarse-grain" electrons in a polarizable classical model may look unusual. However, the separation of motion of nuclei and dummy electrons is required for the polarizable force field to function and achieve high accuracy of the properties as reported. We find that, no matter whether a classical force field or quantum mechanical model is desired, fundamental concepts such as the Born-Oppenheimer approximation need to be observed, whether they are "exactly" implemented or in some analogous form. As the Born-Oppenheimer approximation relates to individual molecules (molecule-wise rather than system-wise), it is also not of concern in the force field that the virtual electrons in gold are of comparable mass as H, C, or O atoms in other molecules because gold is a separate "molecule". The virtual electrons in Au are shielded within the van-der-Waals space of the nucleus, which has a well depth of 3.5 kcal mol^{-1} *versus* only 0.2 kcal mol^{-1} for the electron, so that interaction with atoms in other molecules does not play a significant role other than the intended contribution to polarization. In some sense, the Born-Oppenheimer approximation is qualitatively also applied for H, C, or O in other molecules which have an electron mass of zero as well as no charge (= no inclusion of virtual electrons in typical classical force fields like CHARMM, CVFF, OPLS-AA). Molecular dynamics simulations with the

polarizable model may be considered Car-Parrinello dynamics rather than strictly Born-Oppenheimer dynamics as the polarization is not exactly minimized at each time step.

These choices, after a least squares fit, reproduce (1) the electrostatic attraction of ions in agreement with the classical image potential, (2) the bulk density of gold (determined mainly by σ), (3) the surface energy of gold (determined mainly by the sum of ε values for atom core and dummy electron), (4) and the bulk modulus of gold in agreement with experiment (Table 1). The polarizable force field performs equal or better relative to the neat LJ potential, and mechanical properties are slightly improved (Supplementary Table 1).[7] Therefore, the polarizable potential meets or exceeds the performance of the neat LJ potential and additionally reproduces image potentials and polarization in electric fields. It can be embedded without modification into major classical force fields, as well as into polarizable force fields for proteins and water, to simulate aqueous and biological interfaces. Alternatively, using a hard-wall potential for the metal, a screening function for the interaction with other phases, or an excessively small time step would add further parameters and limit broad applicability.

Validation. The position of the image plane to reproduce the image potential was set equal to the jellium edge, in agreement with experimental data[3] and with widely accepted understanding of metal surfaces (Figure 2).[1, 2, 4] The margin of uncertainty about the position of the image plane is about ±0.3 Å when no experimental data are known. The image plane of gold (111) surface is almost exactly located at the jellium edge, shifted by $x_0 - x_b$ = -0.025 Å inwards (Supplementary Fig. 1a).[3] An alternative reference to reproduce polarizability could also be the static average electric dipole atomic polarizability of an Au atom in the gas phase ($\alpha_V = q^2 /(8\pi\varepsilon_0 k_r)$), however, there are no experimental data available and high level theory calculations suggest 4.7±0.6 $Å^3$ with a significant range of uncertainty.[39, 40]

For the validation of the image potential, a metal slab of at least 6 layers of atoms, 7.5×7.5 nm^2 lateral dimensions, and 10 nm box height was employed to eliminate finite size effects. Smaller boxes (few nm size) can be employed to compute the density, surface (cleavage) energy, and other bulk properties that involve no surfaces with polarization (see ref. [7]). A time step of 1 fs was used in molecular dynamics simulations at room temperature. Larger time steps of 2 fs also conserve the energy. Smaller time steps, *e.g.*, 0.5 fs, can resolve the motion of dummy electrons in greater precision, which is not critical for most applications.

The reproduction of the image potential was initially tested by calculating the difference in electrostatic attraction of a sodium ion between 5 Å and 10 Å distance from the metal surface atomic plane. The force field parameters to be explored to achieve a quantitative match to the image potential were the dummy charge $-q$ (ultimately set to $-1e$) and the bond stretching constant k_{r}. The resulting image potential thereby depends on the ratio q^2 / k_{r}, leaving only one adjustable parameter. Initial Lennard-Jones parameters σ, $\varepsilon_{\mathrm{core}}$, and ε_{e} were needed at this stage for the stability of the model and had no effect on the image potential. Values of k_{r} and q were chosen small enough to prevent potential failure due to excessive Coulomb forces and to allow the use of standard time steps to integrate the equations of motion (rather than reduced time steps). At the same time, large enough values of k_{r} and q (in the fixed ratio q^2 / k_{r}) were chosen to limit random (thermal) motion of the virtual electrons. After this test at two different distances from the metal surface, the entire distance dependence of the image potential was reasonably reproduced and the parameters q and k_{r} required only minor or no further adjustments (Fig. 2a).

Then, the exact Lennard-Jones parameters were determined. The value of σ is very close to the neat LJ potential[7] and determines the density of the metal. The exact choices of $\varepsilon_{\mathrm{core}}$ and ε_{e} modify the surface energy, the short-range distance dependence of interactions with an ion, and

the stability of the potential; they do not affect the image potential which is a Coulomb-only interaction (or almost so). Calculations of density and surface energy were performed using molecular dynamics in the NPT ensemble and in the NVT ensemble, respectively. The parameters σ, $\varepsilon_{\text{core}}$, and ε_{e} were adjusted for the best match of the structure and surface energy to experimental data under standard conditions, consistent with the standards set forth in the INTERFACE force field.[22] k_{r}, σ, $\varepsilon_{\text{core}}$, and ε_{e} were further refined using computations of the electrostatic attraction, density, and surface energy with an iterative least-squares fit.

As a next step, the metal-water interfacial energy and mechanical properties were computed and essentially obtained in best possible agreement with experiment without further parameter adjustments as a result of thermodynamic consistency.[22] Finally, simulations at high temperatures (>1000 K) and in the presence of highly charged Ca^{2+} ions were carried out to verify the stability of the model. Failure by recombination of negatively charged dummy electrons with positively charged metal cations, with neighbor atom cores, or by recombination of anions with atom cores was ruled out. We further verified that the displacement of the dummy electron greater than the atomic radius (~1.45 Å) from the atomic core requires more than the 1st ionization energy (see Supplementary Note 5). A total of over 1000 test calculations were performed for the derivation, validation, and final testing of the force field parameters (less than 500 hundred for derivation). Molecular mechanics and molecular dynamics calculations were carried out with the programs Discover, LAMMPS, GROMACS, and NAMD.

In the evaluation of interaction energies of metal interfaces with water and peptides, Coulomb and van-der-Waals contributions can be specified (Fig. 6). Contributions to the Lennard-Jones energy by the motion of dummy electrons are thereby negligible. The virtual electrons essentially shadow the metal nuclei due to $\sigma_{\text{core}} = \sigma_{\text{e}}$ with very small changes in average distance from the

core (<0.1 Å or <3% of the distance to the next nearest atom cores, respectively). The ε values of the virtual electrons are also less than 10% of those of the atom cores so that the distinguishable contribution of the dummy electrons to the van-der-Waals energy (LJ energy) is very small (<0.3%). The dummy electrons do also not undergo spontaneous polarization on the outer metal surfaces (<10^{-4} Å displacement, if any, see also Supplementary Fig. 7).

This protocol has also been applied to other fcc metals and shows that inclusion of polarizability in this manner is a generally feasible approach. Complete results for a larger set of metals will be communicated separately. The novelty of the models lies mainly in the translation of the electronic structure of the metals into a simple and reliable classical functional form.

Usage. The energy expression and the parameters for the polarizable model are completely stated in Fig. 1c and Fig. 1d. The parameters can be added to any of the force fields AMBER, CHARMM, CVFF, DREIDING, GROMACS/GROMOS, IFF, OPLS-AA (and others that employ the same energy expression) using correct units and default combination rules. Sometimes, equivalent forms of the 12-6 LJ potential are employed:

$$E_{\mathrm{LJ}} = \sum_{\substack{ij \text{ nonbonded} \\ (1,2 \text{ excl})}} \varepsilon_{ij}\left[\left(\frac{\sigma_{ij}}{r_{ij}}\right)^{12} - 2\left(\frac{\sigma_{ij}}{r_{ij}}\right)^{6}\right] = \sum_{\substack{ij \text{ nonbonded} \\ (1,2 \text{ excl})}} 4\varepsilon_{ij}\left[\left(\frac{\sigma_{ij}^{*}}{r_{ij}}\right)^{12} - \left(\frac{\sigma_{ij}^{*}}{r_{ij}}\right)^{6}\right] = \sum_{\substack{ij \text{ nonbonded} \\ (1,2 \text{ excl})}} \left[\frac{A_{ij}}{r_{ij}^{12}} - \frac{B_{ij}}{r_{ij}^{6}}\right] \quad (1)$$

Respective values of σ^*, A, and B can be obtained using the given values of σ and ε in Fig. 1 as $\sigma^* = \frac{\sigma}{2^{1/6}}$, $A = \varepsilon\sigma^{12}$, and $B = 2\varepsilon\sigma^{6}$. Simulations of the metal and complex interfaces can be carried out using common molecular dynamics codes, for example, LAMMPS, NAMD, GROMACS, Discover, Forcite, or DL-POLY.

Supplementary Note 3: Limitations of the Polarizable Model

The free, polarizable electron gas in quantum mechanics corresponds to a structure-less jellium model.[1] In the proposed classical model, the polarization is instead atom-wise and forced to have a spatial structure related to the atomistic structure of the metal. The virtual electrons, although locally flexible, remain bound to each metal atomic core with the approximate ionization energy. The polarizable model does therefore not capture the true electronic structure inside the metal and cannot be used to simulate charge transport and electrical conductivity without further assumptions. It uses an implicit representation of electrons to create the correct surface potential upon applied external fields, but without attempting to represent internal location or mobility of electrons.

In response to an adsorbed ion, the lateral and vertical spread of induced charge in the classical polarizable model is represented by the displacement of dummy electrons from the atomic centers. The dimensions of these lateral and vertical patterns are similar to the dimensions of Friedel fluctuations in quantum mechanical calculations (Supplementary Fig. 1). However, the difference between quantum mechanical features and classical features is that Friedel fluctuations have a periodicity determined by the Fermi wave-vector and are also present when the atomistic structure of the metal is approximated by a structureless jellium. The classical model does not represent continuum or wave-like features. It is particle-based with limited mobility of dummy electrons.

When metal surfaces are grounded, image charges physically arise from the influx of electrons to the metal surface or from the exit of electrons out of the metal surface.[2] Charge inflow or outflow also occurs upon application of an external potential. The classical polarizable model does reproduce the image charge potential, surface, and interfacial interactions of metal nanostructures. However, it does not reproduce the electronic structure and the physical processes that occur inside

the metal. For example, charge accumulation on the surface and internal currents cannot be represented, unless external assumptions are made (Supplementary Fig. 7). However, it does reproduce the effect of external potentials on the surface and related changes in interfacial adsorption, irrespective of whether the metal surfaces or nanostructures are grounded or not. The polarizable model does then not require added parameters for a charge distribution or on-the-fly evaluations of charge distributions. The polarizable atoms adjust internal dipoles in response to external fields on the fly. However, the model is intrinsically not designed to represent charge distributions and currents in the interior of the metal.

The continuum image potential is known to be a good approximation for distances larger than 2.5 Å from the image plane.[3, 4, 41] At closer distance, the response of the polarizable model to positively *versus* negatively charged ions is slightly different. Repulsive potentials are needed to balance increasing attraction, and in this model these are the bond energy of the virtual electrons and the repulsive part of the Lennard-Jones energy. From infinity to a distance of 4 Å, the curves of the image potential for Na^+ ions and Cl^- ions are essentially identical (Fig. 2 and Supplementary Fig. 3). A difference up to 10% emerges at a distance of 3 Å and increases to 20% at 2.5 Å distance. More than half of this difference is compensated by the difference in bond energy of the virtual electrons, however, and therefore does not affect the total energy (Supplementary Fig. 3). At distances below 3 to 5 Å, the LJ potential begins to play a role and leads to curves of total energy that depend on the specific LJ parameters for each ion. The remaining surface corrugation and asymmetry for positively charged *versus* negatively charged ions is rather small. Metal surfaces also respond differently to positively *versus* negatively charged ions as a result of work functions, electronegativity, and chemical properties. An example is the unequal distribution of charges for

sodium ions *versus* chloride ions at close distance from the gold surface in vacuum in DFT calculations (Fig. 3a-d).

The parameterization of the image potential required a choice between the image potential and the atomic polarizability of gold as a reference. Our choice was the image potential (Fig. 2). The image plane at the jellium edge is a simple and widely accepted concept to describe polarization of metals, and experimental data for its location are available.[2, 3, 4] In contrast, experimental data for the polarizability of gold are not available. High-level quantum mechanical calculations at the CASPT2[39] and QCISD(T)[40] levels suggest 4.1 Å^3 and 5.3 Å^3, respectively. Older calculations even suggested of 5.8 Å^3 with a large uncertainty of ±1.5 Å^3.[5] The polarizable model corresponds to $\alpha_V = q^2/(8\pi\varepsilon_0 k_r)$ = 3.3 Å^3. While this value is at the lower end, the computed attraction of ions with the polarizable model in comparison to DFT calculations shows a good match and does not indicate that stronger polarization would be needed (Fig. 3). The results are therefore consistent with the choice of the image plane directly at the jellium edge, supported by experimental data.[3] Further work, such as an extension of this polarizable potential to other fcc and differently structured metals,[42] will show whether the atomic polarizability of the metal could play a stronger role in the validation and how the two concepts could merge. The availability of experimental data for atomic polarizabilities and of further data for the exact location of (hkl) image planes in the future could lead to valuable clarifications.

A quantum mechanical description of the polarization charge density has also been given by Bodrenko et al. and the method was applied to calculate the static polarizability of silver atoms in metallic slabs.[43] A value of 1.5 Å^3 was obtained, which is significantly lower than expected values in a range of 3.0 to 5.5 Å^3.

Supplementary Note 4: Possible Extensions of the Polarizable Model

The representation of internal charge distributions in the metal, such as at electrode surfaces, under applied electric fields, and of currents is feasible using additional assumptions and add-ins in the simulation. (1) The methods by Siepmann and Sprik,[24] Reed et al.,[25] or Merlet et al.[28] can be applied to inject or remove electrons and carry out simulations at constant potential. These methods could be used along with the non-polarizable potential (such as CHARMM-IFF or PCFF-IFF)[7] to avoid double counting physical effects. A benefit of using the non-polarizable LJ parameters is then that bulk and interfacial properties of high quality are obtained. The necessary add-ins would include parameters for the charge distribution (*e.g.* a Gaussian distribution), and changes in the charge distribution must be implemented through added on-the-fly calculations during molecular dynamics to keep the potential constant. (2) The polarizable model can be used to simulate an electrode under an applied potential and associated interfacial processes (Supplementary Fig. 7). The added surface charge can be initially spread across the surface atoms according to physical assumptions of a free electron gas using the concepts described by Siepmann and Sprik,[24] Reed et al.,[25] or Merlet et al.[28] The example of a slab electrode shows that the electrode surface polarizes in response to the surface charge and the inside of the metal remains field free (Supplementary Fig. 7b,c). Subsequent changes of this surface potential due to the dynamics of external ions and molecules do not require updating, or less frequent updating, of the net surface charge distribution as the polarizable model continues to correctly represent induced charges due to an external charge distribution. Updating of the surface charge distribution is necessary, however, when the net potential, *i.e.*, the total net charge, changes, or when the true internal charge distribution in the metal is to be monitored. The displacement of the virtual electrons conveniently indicates the local strength of the electric field in the metal at any point in time (Supplementary Fig. 7a-c). The

advantage of the polarizable model is the elimination, or reduced need, for on-the-fly calculations of charge distributions during molecular dynamics simulations to model the effect of an external potential. Details remain to be explored in follow on studies. (3) Conduction can in principle be modeled by the addition of atomic charges to relevant atoms in the atomistic model, following the desired current/voltage characteristics, and by modification of the charge distribution during molecular dynamics as required. The new polarizable model with an applied potential may be used for this purpose, or the nonpolarizable model with Siepmann-Sprik additions to represent the external potential.

An important application is also the modeling of chemical reactions at gold surfaces. The polarizable force field is inherently a classical, non-reactive force field. Nevertheless, chemical reactions such binding of thiols, atom leaching, and other catalytic processes can be simulated using simple additions in the same way as shown for the non-polarizable model.[44, 45, 46, 47] The first step is knowledge of the changes in bonding from reactants to products, if the actual path of the reaction is less important, or of the mechanisms of the reactions of interest when rate predictions are desired. Such data can be obtained from experiment, from the literature, or from quantum mechanics. Then, triggering criteria for bond breaking, bond formation, or the inclusion of local Morse potentials in the polarizable model for metals (and embedding in force fields such as CHARMM) can be specified in the molecular dynamics code. With such additions, simulations of chemical reactions, predictions of activation energies, and of reaction rates are feasible.

Supplementary Note 5: Calculation of Polarizability and Response to Applied Potentials

The static average electric dipole polarizability for an individual Au atom was calculated for the classical polarizable model and compared to results from high-level quantum mechanical

calculations for an Au atom in the ground state, which range from $4.1\cdot10^{-30}$ m^3 to $5.3\cdot10^{-30}$ m^3.[39, 40] A plot of the computed dipole moment *versus* an applied homogeneous electric field follows the linear relation $\mu(E) = \alpha \cdot E$ and yields α_V = 3.3 (±0.1)$\cdot10^{-30}$ m^3 (Supplementary Fig. 2).

The polarizability α in the model can also be analytically derived. The force F on the dummy electron of charge q as a function of the applied electric field E ($F = q \cdot E$) is compensated by the opposite harmonic force $-k \cdot x$ from the bond stretching potential, which results from the displacement of the dummy electron away from the atom core:

$$F = q \cdot E - k \cdot x = 0. \quad (2)$$

For any strength of the electric field E, the equilibrium between the two forces yields:

$$q \cdot E = k \cdot x. \quad (3)$$

The displacement of the dummy electron x relative to the atomic nucleus in the electric field induces the dipole moment $\mu = q \cdot x$ that is related to the polarizability α of the atom via

$$\mu = \alpha \cdot E. \quad (4)$$

The elongation of the dummy electrons x is thus related to the electric field by

$$x = \frac{\alpha}{q} E, \quad (5)$$

and Supplementary Equation 3 becomes:

$$q \cdot E = k \cdot \frac{\alpha}{q} E \quad (6)$$

$$\alpha = \frac{q^2}{k} = \frac{q^2}{2k_\mathrm{r}}. \quad (7)$$

Supplementary Equation 7 is a simple, universal relation between the square of the charge on the dummy electron and the bond stretching constant k for this type of polarizable model, the ratio of which determines the polarizability. The bond stretching constant k_r in our model (Fig. 1) is thereby half the value of k, since the bond stretching energy is written as $k_r(r_{ij} - r_{0,ij})^2$ rather than $\frac{k}{2}(r_{ij} - r_{0,ij})^2$. Further giving the polarizability in common volume units α_V (α divided by $4\pi\varepsilon_0$), we obtain from Supplementary Equation 7:

$$\alpha_V = \frac{q^2}{8\pi\varepsilon_0 k_r} = 3.32 \cdot 10^{-30}\,\mathrm{m}^3 \tag{8}$$

(k_r was hereby used in atomic rather than molar units, *i.e.*, after division by the Avogadro number N_A). The polarizability of an Au atom in the model 3.3 Å^3 is therefore slightly lower than results from quantum mechanical calculations of 4.7 ± 0.6 Å^3.[39, 40] The choice of parameters for the virtual electron in this model reproduces the image potential at the jellium edge of bulk metal and enables stable simulations (small dummy displacement). If a higher charge q or lower value for k_r were chosen to increase the polarizability of Au (g) atoms, the image plane would effectively shift outward from the jellium edge to be able to reproduce the image potential.

The related polarization energy, or work W performed by an electric field E on a polarizable gold atom can be described by integration of the forces over the displacement x using Supplementary Equation 3:

$$W = \int_{\substack{E=0\\x=0}}^{\substack{E=E_m\\x=l}} q \cdot E(x) \cdot dx = \int_{\substack{E=0\\x=0}}^{\substack{E=E_m\\x=l}} k \cdot x(E) \cdot dx \tag{9}$$

$$W = \int_{\substack{E=0\\x=0}}^{\substack{E=E_m\\x=l}} \frac{q^2}{\alpha} \cdot x \cdot dx = \int_{\substack{E=0\\x=0}}^{\substack{E=E_m\\x=l}} k \cdot x \cdot dx \tag{10}$$

The result can be expressed in various equivalent forms:

$$W = \frac{q^2 l^2}{2\alpha} = \frac{\mu_\mathrm{m}^2}{2\alpha} = \frac{\mu_\mathrm{m} E_\mathrm{m}}{2} = \frac{\alpha E_\mathrm{m}^2}{2} = \frac{k l^2}{2}\,. \tag{11}$$

Thereby, l is the final displacement, μ_m the final dipole moment, and E_m the final electric field strength.

The simulations to calculate the atomic polarizability involved placement of a polarizable Au atom in an electric field generated between two oppositely charged plates. Two setups were tested including (i) two charged plates of 2.31×2.50 nm^2 area, spaced 3 nm apart (2×80 atoms), in a box with 3D periodic boundary conditions and a total box height of 1000 nm, and (ii) two larger charged plates of finite size of 23.1×25.0 nm^2 area, spaced 3 nm apart (2×8000 atoms), in an open box without periodic boundaries. The charged plates consisted of a layer of fixed atoms (without virtual electrons) that were uniformly charged with 0.01e, 0.1e, and 1e, respectively, positive on one plate and negative on the other. This setup created a uniform charge density Q/A, electric field $E = \frac{Q}{\varepsilon_0 A}$, and voltage $U = El = \frac{Ql}{\varepsilon_0 A}$. The voltages were 7.47 V, 74.7 V, and 747 V accordingly. The plates mimicked a parallel plate capacitor with a homogeneous, constant electric field between the two plates. A similar setup can also be used to study the response of a bulk metal interface under a constant external potential, *e.g.*, an electrode-solution interface with adsorbed proteins or enzymes in a biosensor.

The calculation protocol was as follows: (1) Fix the atoms constituting the plates at z = 0.0 and at z = 3.0 nm with charges $-q$ and $+q$, cell height 1000 nm for high q (±1.0e), and lower height of 30 nm for low q (±0.01e) to avoid artifacts in Ewald summation, (2) add the Aue atom with fixed Au position and flexible dummy electron in the middle between the plates and carry out energy minimization to displace the dummy electron, (3) record dummy displacement l, (4) then allow flexibility of all atoms and calculate single point energy E (with Aue), (5) remove Aue atom and calculate single point energy again with all plate atoms flexible E (without Aue), (6) determine reference energies of the plates without charge (confirmed to be the same with and without Aue added).

For each charge setting, the electric field E is known (2.5, 25, and $250 \cdot 10^9$ V m^{-1}), and the induced dipole moment on the polarizable Au atom was calculated $\mu = q \cdot l$ (q being the core charge of Aue and l the displacement). The curve $\mu = f(E)$ was perfectly linear and the polarizability α calculated as $\alpha = \mu / E = q \cdot l / E$ (Supplementary Fig. 2). The result was α_V = 3.3±0.1 $Å^3$ for the periodic plates, matching with the analytical result from Supplementary Equation 9. The open plates lead to α_V = 3.0±0.1 $Å^3$ as the finite area of the plates results in a slightly weaker electric field strength than infinitely large plates.

The energy, or work of stabilization W related to the formation of the dipole was obtained as:

$$W = [E(\text{plates charged with Aue}) - E(\text{plates non-charged with Aue}] - [E(\text{plates charged no Aue}) - E(\text{plates non-charged no Aue}] \quad (12)$$

$$W = E(\text{plates charged with Aue}) - E(\text{plates charged no Aue}) \quad (13)$$

A difference in the derivation of the energies in this case, compared to Supplementary Equations 9 to 11, is a constant electric field E throughout the development of the core-electron dipole.

Supplementary Equations 9 to 11 assume a changing (gradually increasing) electric field and are thus no longer valid. The electrical energy of creating the dipole in the Aue atom now is:

$$W_{\text{Coulomb}} = -\int_{x=0}^{x=l} q \cdot E \cdot dx = -q \cdot E \cdot l = -\mu \cdot E = -\alpha \cdot E^2 \quad (14)$$

without a factor ½. The mechanical energy of stretching the dummy electron remains the same

$$W_{\text{Bond}} = -\int_{x=0}^{x=l} k \cdot x \cdot dx = \frac{1}{2} kl^2 , \quad (15)$$

and the energy balance in the simulation is then

$$W_{\text{Coulomb}} = -2 \cdot W_{\text{Bond}} = -kl^2 \quad (16)$$

and

$$W_{\text{Total}} = W_{\text{Coulomb}} + W_{\text{Bond}} = 1/2 \cdot W_{\text{Coulomb}} = -W_{\text{Bond}} . \quad (17)$$

In other words, the Coulomb energy is a negative value, due to formation of the stabilizing dipole in the *E* field, and it is twice as large as the bond stretching energy. The bond stretching energy remains a positive energy contribution. The polarizabilities calculated from Supplementary Equation 14, $\alpha = -W_{\text{Coulomb}} / E^2$, or $\alpha_V = -W_{\text{Coulomb}} /(4 \cdot \pi \cdot \varepsilon_0 \cdot E^2)$, are α_V = 3.3±0.1 Å^3 for the periodic plates and α_V = 2.6±0.1 Å^3 for the open plates (the true *E* field is then slightly weaker than the ideal *E* field used in the calculation), consistent with the results from dipole moments.

Moreover, the displacement of the dummy electron reaches the atomic radius of Au (~1.44 Å) when the Coulomb energy (Supplementary Equation 14) approaches the first ionization energy of gold of ~890 kJ mol^{-1}. This result confirms the stability of the dummy electron and adds to the

physical justification of the model. The response of a metal slab to an applied potential was also tested and found to be fully reversible (Supplementary Fig. 7).

Supplementary Note 6: Gold-Ion Interfaces in Vacuum

This note discusses details of the interactions of the ions with the surface and local surface deformations.

Interaction of the Ions with the Surface. Specific van-der-Waals diameters (σ) and dispersive energies (ε) in the Lennard-Jones potential for the Na^+ and Cl^- ions individualize the energy profiles at distances <4 Å from the metal (Supplementary Fig. 3). The Na^+ ion is smaller than the Cl^- ion and approaches the gold surface more closely in the polarizable model, leading to stronger Coulomb attraction and an equilibrium distance of 1.86 Å from the top atomic layer. The larger Cl^- ion does not approach the surface as closely and experiences less attractive polarization. The equilibrium distance is 2.81 Å. Differences in the energy profiles obtained via energy minimization (temperature = 0 K) and via NVT molecular dynamics simulations (temperature = 298 K) are <0.5% in equilibrium distance and <1% in binding energies. The values are essentially the same due to the high energies involved in adsorption (>50 kT). Small differences in kinetic energy are negligible.

The response of the polarizable model is similar to positive and negative charges up to approximately 2.5 Å distance from the jellium edge, even though negatively charged dummy electrons are employed (Supplementary Fig. 4). The electrostatic attractive energy jumps to large negative values at a distance of ~2 Å from the jellium edge for q = +1.0e, and similarly at a distance of ~1 Å from the jellium edge for q = -1.0e (indicated by arrows in Supplementary Fig. 4). The total interaction energy is almost the same depending on the equilibrium distance of the specific

ion from the surface (see Fig. 2b in the main text). It is known that a match of the attraction energy to the classical continuum model is only physically meaningful for distances from the jellium edge between ~2.5 Å and $+\infty$ as closer distances overestimate the attraction and require other descriptions.[3, 4] The curves for electrostatic attraction remain nearly the same independent of the chemical identity of the ions (for example, Na^+, K^+ for single positively charged ions and Cl^-, Br^- for single negatively charged ions) as only the contribution of Coulomb energy is assessed. The vertical thickness of the metal slab was 21.1 Å (9 atomic layers) to represent a bulk metal surface in the computation of surface interaction energies, and the box height was 100 nm to eliminate effects from 3D periodic boundary conditions in the vertical direction.

Local Surface Deformation. When Na^+ ions are close to the surface <4 Å, the dummy electrons are pulled out from the nearest atom cores up to ~0.3 Å and atom cores rise 0.05 to 0.1 Å above the normal surface level. When the closest Au atom cores are pulled out by 0.1 Å from the surface at an initial Na^+ distance of 2.8 Å, the true distance was determined relative to the actual outer surface, *i.e.*, 2.7 Å in this example (see also Fig. 2b, Supplementary Figs. 3 and 6). At distances shorter than equilibrium distance, the Au atom cores are locally pushed inwards. For example, at a very short initial distance of 1.0 Å, the nearest Au atom cores may displace 0.25 Å inwards and the actual distance is then ~1.25 Å. Local deformation/compression of the surface occurs to counterbalance higher energies.

Similarly, the nearest Au atom cores are slightly pulled out from the surface atomic plane by Cl^- ions at distances <4 Å with an upward displacement up to 0.1 Å. At the equilibrium distance of Cl^- ions, gold atom cores are approximately back in equilibrium positions (+0.02 Å) and dummy electrons are pushed inwards by about 0.2 Å. Below equilibrium distance, the atom cores are pushed inwards, for example, by about 0.1 Å at 2.6 Å distance and up to 0.3 Å at 2.2 Å distance.

The distance of the ion from the surface was always recorded relative to the vertical position of the nearest surface atom cores, *i.e.*, somewhat shorter or larger than the vertical distance to the unperturbed metal atoms in the top atomic layer. The uncertainty in reported distances is <0.01 Å for distances larger than 5 Å from the surface and increased to about ±0.02 Å for distances near and shorter than the equilibrium distance.

Summary of Equilibrium Distances and Energies. The equilibrium distances and energies according to the force field are 1.86 Å, -58.5 kcal mol^{-1} for Na^+ ions, and 2.81 Å, -34.0 kcal mol^{-1} for Cl^- ions (Fig. 2b). Interestingly, the equilibrium distances differ from DFT values of 2.5 Å for Na^+ ions and 2.1 Å for Cl^- ions (Fig. 3e, f).. Partial covalent bonding of Cl^- ions to the gold surface may explain the short distance for Cl^- ions. The comparison of equilibrium adsorption energies from the force field relative to DFT values is difficult due to the uncertain position of the zero energy in DFT (Fig. 3e,f). The unphysical shape of the DFT curve at larger distance (using the PBE functional) is related to a strong electron delocalization error and an inaccurate description of the long range van der Waals interaction.[48, 49, 50, 51, 52]

Supplementary Note 7: Range of Induced Charges and Required Dimensions of Surface Models

The thickness of the metal slab must be at least six atomic layers (>12 Å) to mimic properties of the bulk metal due to the range of van-der-Waals and locally compensated electrostatic interactions. The derivation of the force field parameters was carried out with surface models of a thickness of nine atomic layers (>21 Å) to eliminate this source of error without doubt.

A less obvious factor are the necessary lateral dimensions and height of 3D periodic simulation boxes. The electric monopoles (non-neutralized ions) in the 3D periodic system cause long range

interactions with periodic images and patterns of surface dipoles on the metal slabs that are uncommon in electroneutral systems. As a result, computed Coulomb interaction energies are influenced by the lateral box dimensions as well as by the box height, which must exceed a certain threshold to represent a continuum system and allow quantitative comparisons with the classical image potential (Supplementary Fig. 4 and Supplementary Table 2). Uncompensated electric monopoles such as Na^+ and Cl^- ions in vacuum require, as a safe estimate, box dimensions of 100×100×1000 Å that provide enough space vertically and laterally to reduce long-range interactions with periodic images and to evaluate image charge potentials with less than 1% deviation from the continuum value. Such box dimensions, which include many thousand metal atoms in the surface slab are also beyond the reach of quantum mechanical methods and only feasible by classical simulations. Standard Ewald summation and PPPM/PME methods can be used to compute the electrostatic interactions including the image charge potential as routinely done in molecular dynamics simulations. A slab correction for the Ewald method can be applied to reduce computing time[53] but is not required to increase the accuracy.

In detail, interestingly, lateral box dimensions and box height have opposite effects on the computed Coulomb energy (Supplementary Table 2). (1) In boxes of small lateral dimensions of 14.4×15.0 $Å^2$, the dummy electrons on the metal surface build a surrounding field of dipoles limited to three or two atomic layers in *x* and *y* direction on the surface, respectively, in response to an adsorbed ion before meeting a neighbor cell. This confinement severely restricts the response to charges near the surface (Supplementary Fig. 1e,f) and the Coulomb energy is underestimated (-19.2 kcal mol^{-1} *versus* -44.7 kcal mol^{-1} for a large system in Supplementary Table 2 and Supplementary Fig. 4), assuming a large box height of 1000 Å. (2) In contrast, lateral box dimensions of 101×100 $Å^2$ allow the image charge to spread over 18 or 10 surface atomic layers

in x and y direction, respectively, before reaching a neighbor cell. This size, or already the size of 72×75 $Å^2$, allows near-equilibrium spreading of the image charge in response to the single charged ion, eliminates finite size effects due to periodic neighbors, and results in convergence of Coulomb energy (-44.7 kcal mol^{-1} in Supplementary Table 2 and Supplementary Fig. 4). (3) A shorter box height of 100 Å *versus* 1000 Å also shows a strong effect, especially when lateral dimensions are small, such as 14.4×15 $Å^2$ (-56.7 kcal mol^{-1} *versus* -19.2 kcal mol^{-1} in Supplementary Table 2). The proximity of many lateral and vertical neighbors then strongly changes the Coulomb energy. While the repulsion between the ion and its vertical periodic images is constant at a large distance from the surface (*e.g.* >30 Å), it diminishes when the ions approach the surface. The reason is the buildup of a large array of oriented dipoles of nearby dummy electrons and atom cores that distributes the charge of the adsorbed ion over several layers of metal atoms both laterally and vertically; the charge is "smeared out". This effect increases the dielectric constant of the metal slab, which separates the original ion and its vertical periodic images. In effect, the repulsion between the ion and its vertical periodic images is reduced, which reflects in an increase in attraction at near distance (-56.7 kcal mol^{-1} in Supplementary Table 2). (4) A small box height, paired with small surface area, can lead to a partial cancellation of errors, for example, it may invert the expected trend in attraction for small *versus* large surface area (-56.7 kcal mol^{-1} *versus* 46.06 kcal mol^{-1} in Supplementary Table 2). For large surface area, the density of vertical images is drastically reduced ($1/25^{th}$ in Supplementary Table 2) and the effect of box height weakens, too. Therefore, systems of 72×75 $Å^2$ or larger lateral dimension yield nearly identical results for small and large box height (-46.1 and -44.7 kcal mol^{-1} for 100 Å and 1000 Å, respectively, in Supplementary Table 2). The attraction still tends to be slightly stronger for small box height.

In summary, it is critical to use sufficiently large and high boxes to eliminate finite size effects in the presence of electric monopoles (free ions) in vacuum. Boxes larger than a minimum size of 80×80×100 $Å^3$ are essentially error-free and recommended for force field development. Box sizes beyond 100×100×1000 $Å^3$ lead to negligible errors (<1%). For smaller lateral dimensions and box heights, results are generally not reliable even though a partial cancellation of errors can occur (*e.g.* -44.7 *versus* -56.3 kcal mol^{-1} in Supplementary Table 2).

Supplementary Note 8: Gold-Aqueous Interfaces

The inclusion of dummy electrons in the metal potential does not change the interaction of water molecules with the metal surface significantly except for single molecules. The binding energies of single, isolated water molecules are stronger (further below zero) when induced charges are taken into account. The effect of polarization is minor for a water monolayer or bulk water adsorbed onto the surface. Preferences toward epitaxial sites are found with polarizable and with non-polarizable models, which is especially visible on the (100) surface.

A significant effect of polarization on the adsorption of single water molecules is seen as then induced charges can spread out over several atomic layers across the gold surface similar as upon adsorption of ions. The adsorption energies are -5.5 *versus* -3.9 kcal mol^{-1} on Au (100) surfaces with the polarizable *versus* the nonpolarizable model, associated with the preferred location of oxygen atoms in water molecules at (100) epitaxial sites and the close proximity of these sites four polarizable neighbor gold atoms (Table 2, Supplementary Movies 1 and 2). The effect of polarization is less on the Au (111) surface that provides less area and only three atomic neighbors per epitaxial site (Table 2, Supplementary Movies 3 and 4). Overall, polarization increases and somewhat equalizes adsorption on both facets at the same time. Experimental data for

physisorption of water on gold indicate ~-5 kcal mol^{-1} adsorption energy,[54] which is very close to the result with the force field. The original adsorption data indicate monolayer formation at 10% relative humidity at 25 °C. The corresponding adsorption energy was obtained using the Langmuir relation for surface coverage $\theta = Kp/(1+Kp)$ for several data points (including $p = 0.1p_0 = 0.3$ kPa = 0.003 atm) via $\Delta G = -RT\ln K$. Chemisorption is also possible at later stages, and chemisorption energies were reported much stronger as -25 kcal mol^{-1} [55] and as -35 kcal mol^{-1}.[56] Chemisorption cannot be directly probed with the classical simulation, however, it can become feasible after implementing extensions that allow bond breaking.

In the condensed phase such as water monolayers, polarization no longer enhances adsorption in a significant way (Fig. 4a, b). Different from the observations for single molecules or ions (Fig. 4a), dipoles in neighbor water molecules are then very close and permit no consistent polarization across multiple atomic layers in the metal (Fig. 4b). Therefore, polarization is randomized and near zero. Facet-specific differences in the adaptation to epitaxial sites and in the formation of hydrogen bonds are apparent, whereby the polarizable and the non-polarizable force field show the same trends (Supplementary Movies 5 to 8). Experimentally determined adsorption energies for a water monolayer on polycrystalline gold surfaces are -13 kcal mol^{-1} [56] and -15.5 kcal mol^{-1}.[57] The computed values in the range -12.8 to 14.3 kcal mol^{-1} are in excellent agreement (Fig. 4a).

Hydration and interfacial energies for bulk water are also nearly identical using the polarizable and the non-polarizable model. Dipoles of molecules in bulk water are too small and random in orientation to induce significant charges in the metal, and lateral space for an unperturbed development of image charges is not available. As a result, no net attraction by polarization is added (Table 2). Water is slightly more attracted to (100) surfaces than to (111) surfaces due to a good fit via both soft epitaxy and a suitable spacing to form strong hydrogen bonds (see

Supplementary Movies 5 and 6 for the water monolayer). Experimental data for bulk gold-water interfaces are scarce. Computed gold-water interfacial tensions of ~1150 mJ m^{-2} agree with experimentally observed contact angles of 0° that set an upper limit of the gold-water interfacial energy <1410 mJ m^{-2} according to the Young equation but do not specify a particular value (Table 2).[7, 58] Quantitative agreement at the monolayer scale, as shown above, suggests that simulations are ±5% accurate. Agreement of computed adsorption energies and conformations of organic molecules with experimental and ab-initio data (<10% deviation),[59, 60, 61] as well as detailed predictions of facet selectivity, crystal growth, and catalytic reactivity have also been shown using the nonpolarizable models.[45, 62, 63, 64] These data, mostly for neutral molecules or molecules with few ionic groups adsorbed to metal surface, strongly support the polarizable model as well. Previous simulations also suggested correlations between the in-plane structure of the liquid and the in-plane structure of the charge distribution,[65, 66] which are seen here at the DFT (Fig. 4c) and force field levels of simulation.

The non-polarizable model is not accurate, however, when ions are added into the solution (Fig. 5b) or when significant external potentials are applied. Preferred positions on the surface, the free energy profile, and dynamics of the ions are influenced by polarization. These effects are anticipated to scale up as the complexity of the system increases and multiple chemical species are involved, and the use of the polarizable model then leads to more reliable results.

Supplementary Note 9: Binding of Charged Peptides to Gold Surfaces in Aqueous Solution

Peptide conformations and their impact on binding strength were analyzed using NPT molecular dynamics simulations with the polarizable model and with the nonpolarizable model (Fig. 6 and Supplementary Fig. 10). The DYKDDDDK peptide easily undergoes changes in conformations

and such conformational changes on the surface as well as in solution significantly affect the binding energy. Polarization affects key features such as binding strength and Coulomb *versus* van-der Waals contributions. Details of the interaction with each surface are described in the following.

Aue (111) Surface. When the peptide is away from the surface in solution, it samples through various conformations and maintains the salt bridge K3-Cterm(K8) as well as temporarily an Nterm-D7 bridge. Na^+ ions travel throughout the solution and barely contact the surface (<3% of time). Na^+ ions were found to remain close to the peptide taking into account periodic images.

On the surface, the peptide is fully in direct contact and shows a soft epitaxial match (Fig. 6a). A K3-D7 salt bridge is formed. Na^+ ions travel throughout the solution and only short contacts with the surface were found (<5% of time). The adsorption energy is -45.5±2 kcal mol^{-1} (electrostatic contribution +29 kcal mol^{-1}). In case of more Na^+ contact with the surface of 15% and 30% of time, as seen in some trajectories, the adsorption energy increases by +10 to +20 kcal mol^{-1}, showing that surface contact of sodium ions is not greatly favorable.

The polarizable Aue (111) surface shows highly unfavorable electrostatic contributions to adsorption of the peptide, related to the loss of Au-water contacts with comparatively high atomic charges on O and H atoms (-0.82e and +0.41e). The loss in Coulomb attraction is overcompensated by the gain in van-der-Waals interactions of the peptide with the metal by soft epitaxy.[60]

Au (111) Surface. When the peptide is away from the surface in solution, it assumes a K3-D7 bridge, an Nterm-D6 bridge, and samples through various other conformations including D4-K8, Nterm-D5, and D6-K8. Na^+ ions travel throughout the solution and have <5% of time contact with the surface, of which direct contact is typically <1%.

On the surface, the entire peptide is in direct contact all the time (Fig. 6b). A stable K3-D7 salt bridge was observed, with the D1 side chain oriented upright, and a great epitaxial fit identified (Fig. 6). Sodium ions remained well distributed in solution and 25% of time near the surface, although marginally in direct contact (<2% of time). The adsorption energy is -41.5±1 kcal mol^{-1} (electrostatic contribution -68 kcal mol^{-1}). When a D5-K8 bridge was formed with the D1 side chain oriented upright, the adsorption energy was reduced to -30 kcal mol^{-1}. The energy of -30 kcal mol^{-1} with a D5-K8 bridge was further reduced to -26 kcal mol^{-1} when one sodium ion travels onto the opposite metal surface in the periodic model system.

The nonpolarizable Au (111) model shows favorable electrostatic contributions to adsorption of the peptide, related to the release of water from the surface upon binding of peptide and the gain of numerous electrostatically favorable hydrogen bonds in solution (~20 H_2O molecules per peptide × -3 kcal mol^{-1} per one added hydrogen bond ≈ -60 kcal mol^{-1}). This example highlights the importance of fine detail of conformations on adsorption. A less equilibrated structure was reported in a previous publication with an adsorption energy of -20.5±3.5 kcal mol^{-1}.[67] The dynamics of the sodium ions differs from that using the polarizable model.

Aue (100) Surface. When the peptide DYKDDDDK is away from the surface in solution, a salt bridge between K3 and Cterm(K8) is observed most of the time in equilibrium. Na^+ ions travel throughout the solution. Contact with the gold surface occurs <5% of the time, most of the time with a remaining water interlayer in between. Rarely did sodium ions directly approach the surface (<2% of time).

On the surface, the peptide maintains a conformation with a salt bridge between K3 and Cterm(K8) and remains separated by a water layer all the time (Fig. 6c). Na^+ ions move throughout the solution phase and contact the surface less than 10% of time, more often than without

polarization. The adsorption energy is -13±1 kcal mol^{-1} (electrostatic contribution -5.5 kcal mol^{-1}). If another salt bridge forms on the surface, or no salt bridge at all, the adsorption energy increases to ±0 to +7 kcal mol^{-1}. If the peptide is brought into full direct contact with the surface, the adsorption energy increases to +29±2 kcal mol^{-1} (note lower magnitude of this change compared to the nonpolarizable model).

Therefore, about -11 or -12 kcal mol^{-1} stabilization energy is derived from the K3-Cterm(K8) salt bridge upon surface binding, and some stabilization as well in solution. The electrostatic contribution to adsorption with the polarizable model (-5.5 kcal mol^{-1}) is higher than with the nonpolarizable model (+1 kcal mol^{-1}) even though the total adsorption energy is essentially the same.

Au (100) Surface. When the peptide is away from the surface in solution, K3 and D7 form a steady salt bridge for >90% of time in equilibrium. Na^+ ions travel throughout the system and contact with the surface occurs <10% of time, most of the time with a water interlayer and sometimes without a water interlayer. Alternatively, D4-K8 and Nterm-D7 bridges were stable in solution and, at times, a D6-K8 bridge was observed.

On the surface, the peptide remains separated by a water monolayer and changes slightly conformations (Fig. 5d). The peptide assumes a salt bridge between K3 and Cterm (K8) that is essential for stabilization. Sodium ions remain in solution and are rarely in contact with the surface (<5% of time), either separated by a water interlayer or in direct contact. The adsorption energy is -12±2 kcal mol^{-1} (electrostatic contribution +1 kcal mol^{-1}). When there is no salt bridge and the peptide is stretched out on the surface separated by a water monolayer, the adsorption energy increases to 0.0±1 kcal mol^{-1}. If the peptide is partially or fully in contact with the surface, the adsorption energy increases to +20/+40 kcal mol^{-1} (note the larger change compared to the

polarizable model). The dynamics of the sodium ions on the (100) surface is notably different compared to the polarizable model.

Summary of Findings. The polarizable model is overall more accurate than the nonpolarizable model, and differences in total binding energies are on the order of noncovalent intermolecular interactions. In comparison to isolated ions in the gas phase, the impact of attractive polarization is an order of magnitude less even for this considerably ionic system in the solution phase. Nevertheless, the polarization interactions make a contribution as the observed changes similar to the strength of hydrogen bonds could induce differences in protein folding, catalytic activity, or crystal growth. Perhaps most significantly, electrostatic *versus* van-der-Waals contributions to binding change entirely. The results agree with earlier a-posteriori calculations of the image potential on Au (111) surfaces that predicted about 10% stronger adsorption when induced charges are included.[2] However, an increase in adsorption on the (100) surface due to a water interlayer could not be confirmed in this more accurate model as the hydration shell of ions is maintained most of the time in the presence of the polarizable metal surface. The new polarizable model supersedes a-posteriori methods[2] by including induced charges instantaneously, in higher accuracy, and no longer requiring dedicated scripting and post-processing.

It is also found that peptide adsorption and assembly critically depend on conformations, and that the conformation search is a challenge even when using advanced replica exchange and annealing techniques. A series of simulations informed by the analysis of visual (structure) and energy trends in preceding replicas was necessary to obtain trustworthy results. Exchange of equilibrium conformations on the surfaces using the polarizable and nonpolarizable models in addition to random replica exchanges and independent conformation sampling reduced the

uncertainty in binding energies to about ±1 kcal mol^{-1}, which would otherwise remain as high as ±5 kcal mol^{-1}.

Polarization on the metal surface had notable impact on the adsorption of sodium ions from aqueous solution. While the hydration shell of the ions provides more stabilization than direct contact with the metal surface supported by induced charges, the polarizable model showed more contact on (111) and (100) surfaces than the non-polarizable model. Even so, sodium ions approached the surfaces infrequently (<10% of time) and a water interlayer was present for most of this percentage of time.

Supplementary References

1. Lang ND, Kohn W. Theory of Metal Surfaces: Induced Surface Charge and Image Potential. *Phys Rev B* **7**, 3541-3550 (1973).

2. Heinz H, Jha KC, Luettmer-Strathmann J, Farmer BL, Naik RR. Polarization at Metal-Biomolecular Interfaces in Solution. *J R Soc Interface* **8**, 220-232 (2011).

3. Smith NV, Chen CT. Distance of the Image Plane from Metal Surfaces. *Phys Rev B* **40**, 7565-7573 (1989).

4. Finnis MW. The Interaction of a Point Charge With an Aluminum (111) Surface. *Surf Sci* **241**, 61-72 (1991).

5. Lide DR. *CRC Handbook of Chemistry and Physics, 96th ed.* CRC Press (2015).

6. *Single Crystal Elastic Constants and Calculated Aggregate Properties: A Handbook*. 2nd ed.; Simmons, G., Wang, H., eds.; MIT Press (1971).

7. Heinz H, Vaia RA, Farmer BL, Naik RR. Accurate Simulation of Surfaces and Interfaces of Face-Centered Cubic Metals Using 12−6 and 9−6 Lennard-Jones Potentials. *J Phys Chem C* **112**, 17281-17290 (2008).

8. Drude P. Zur Elekronentheorie der Metalle. I. Teil. *Ann Phys* **306**, 566-613 (1900).

9. Drude P. Zur Elektronentheorie der Metalle; II. Teil. Galvanomagnetische und Thermomagnetische Effecte. *Ann Phys* **308**, 369-402 (1900).

10. Lorentz HA. The Motion of Electrons in Metallic Bodies, I. *Proc K Ned Akad Wet* **7**, 438-453 (1905).

11. Lorentz HA. The Motion of Electrons in Metallic Bodies, II. *Proc K Ned Akad Wet* **7**, 585-593 (1905).

12. Lorentz HA. The Motion of Electrons in Metallic Bodies, III. *Proc K Ned Akad Wet* **7**, 684-691 (1905).

13. Dick BG, Overhauser AW. Theory of the Dielectric Constants of Alkali Halide Crystals. *Phys Rev* **112**, 90-103 (1958).

14. Mitchell PJ, Fincham D. Shell-Model Simulations by Adiatabic Dynamics. *J Phys Condens Mat* **5**, 1031-1038 (1993).

15. Yu HB, van Gunsteren WF. Accounting for Polarization in Molecular Simulation. *Comput Phys Comm* **172**, 69-85 (2005).

16. Lamoureux G, Roux B. Modeling Induced Polarization with Classical Drude Oscillators: Theory and Molecular Dynamics Simulation Algorithm. *J Chem Phys* **119**, 3025-3039 (2003).

17. Lemkul JA, Huang J, Roux B, MacKerell AD. An Empirical Polarizable Force Field Based on the Classical Drude Oscillator Model: Development History and Recent Applications. *Chem Rev* **116**, 4983-5013 (2016).

18. Cochran W. Lattice Dynamics of Ionic and Covalent Crystals. *CRC Crit Rev Solid State Sci* **2**, 1-44 (1971).

19. Kunz APE, van Gunsteren WF. Development of a Nonlinear Classical Polarization Model for Liquid Water and Aqueous Solutions: COS/D. *J Phys Chem A* **113**, 11570-11579 (2009).

20. van Maaren PJ, van der Spoel D. Molecular Dynamics Simulations of Water With Novel Shell-Model Potentials *J Phys Chem B* **105**, 2618-2626 (2001).

21. Heinz H, Suter UW. Atomic Charges for Classical Simulations of Polar Systems. *J Phys Chem B* **108**, 18341-18352 (2004).

22. Heinz H, Lin T-J, Mishra RK, Emami FS. Thermodynamically Consistent Force Fields for the Assembly of Inorganic, Organic, and Biological Nanostructures: The INTERFACE Force Field. *Langmuir* **29**, 1754-1765 (2013).

23. Mahoney MW, Jorgensen WL. A Five-Site Model for Liquid Water and the Reproduction of the Density Anomaly by Rigid, Nonpolarizable Potential Functions. *J Chem Phys* **112**, 8910-8922 (2000).

24. Siepmann JI, Sprik M. Influence of Surface-Topology and Electrostatic Potential on Water Electrode Systems. *J Chem Phys* **102**, 511-524 (1995).

25. Reed SK, Lanning OJ, Madden PA. Electrochemical Interface Between an Ionic Liquid and a Model Metallic Electrode. *J Chem Phys* **126**, 084704 (2007).

26. Willard AP, Reed SK, Madden PA, Chandler D. Water at an Electrochemical Interface—A Simulation Study. *Faraday Discuss* **141**, 423-441 (2009).

27. Willard AP, Limmer DT, Madden PA, Chandler D. Characterizing Heterogeneous Dynamics at Hydrated Electrode Surfaces. *J Chem Phys* **138**, 184702 (2013).

28. Merlet C, *et al.* On the Molecular Origin of Supercapacitance in Nanoporous Carbon Electrodes. *Nat Mater* **11**, 306-310 (2012).

29. Onofrio N, Strachan A. Voltage Equilibration for Reactive Atomistic Simulations of Electrochemical Processes. *J Chem Phys* **143**, 054109 (2015).

30. Breitsprecher K, Szuttor K, Holm C. Electrode Models for Ionic Liquid-Based Capacitors. *J Phys Chem C* **119**, 22445-22451 (2015).

31. Tyagi S, Suzen M, Sega M, Barbosa M, Kantorovich SS, Holm C. An Iterative, Fast, Linear-Scaling Method for Computing Induced Charges on Arbitrary Dielectric Boundaries. *J Chem Phys* **132**, 154112 (2010).

32. Iori F, Corni S. Including Image Charge Effects in the Molecular Dynamics Simulations of Molecules on Metal Surfaces. *J Comput Chem* **29**, 1656-1666 (2008).

33. Wright LB, Rodger PM, Corni S, Walsh TR. GoIP-CHARMM: First-Principles Based Force Fields for the Interaction of Proteins with Au(111) and Au(100). *J Chem Theor Comput* **9**, 1616-1630 (2013).

34. Corni S, Hnilova M, Tamerler C, Sarikaya M. Conformational Behavior of Genetically-Engineered Dodecapeptides as a Determinant of Binding Affinity for Gold. *J Phys Chem C* **117**, 16990-17003 (2013).

35. Cicero G, Calzolari A, Corni S, Catellani A. Anomalous Wetting Layer at the Au(111) Surface. *J Phys Chem Lett* **2**, 2582-2586 (2011).

36. Hoefling M, Iori F, Corni S, Gottschalk KE. Interaction of Amino Acids With the Au(111) Surface: Adsorption Free Energies from Molecular Dynamics Simulations. *Langmuir* **26**, 8347-8351 (2010).

37. Pensado AS, Padua AAH. Solvation and Stabilization of Metallic Nanoparticles in Ionic Liquids. *Angew. Chem.-Int. Ed.* **50**, 8683-8687 (2011).

38. Atkins PW, Friedman RS. *Molecular Quantum Mechanics, 3rd ed.* Oxford University Press (1997).

39. Roos BO, Lindh R, Malmqvist PA, Veryazov V, Widmark PO. New Relativistic ANO Basis Sets for Transition Metal Atoms. *J Phys Chem A* **109**, 6575-6579 (2005).

40. Schwerdtfeger P, Bowmaker GA. Relativistic Effects in Gold Chemistry. 5. Group 11 Dipole Polarizabilities and Weak Bonding in Monocarbonyl Compounds. *J Chem Phys* **100**, 4487-4497 (1994).

41. Jha KC, Liu H, Bockstaller MR, Heinz H. Facet Recognition and Molecular Ordering of Ionic Liquids on Metal Surfaces. *J Phys Chem C* **117**, 25969-25981 (2013).

42. Xu R, *et al.* Three-Dimensional Coordinates of Individual Atoms in Materials Revealed by Electron Tomography. *Nat Mater* **14**, 1099-1103 (2015).

43. Bodrenko IV, Sierka M, Fabiano E, Della Sala F. A Periodic Charge-Dipole Electrostatic Model: Parametrization for Silver Slabs. *J Chem Phys* **137**, 134702 (2012).

44. Ramezani-Dakhel H, Bedford NM, Woehl TJ, Knecht MR, Naik RR, Heinz H. Nature of Peptide Wrapping onto Metal Nanoparticle Catalysts and Driving Forces for Size Control. *Nanoscale* **9**, 8401-8409 (2017).

45. Bedford NM, *et al.* Elucidation of Peptide-Directed Palladium Surface Structure for Biologically Tunable Nanocatalysts. *ACS Nano* **9**, 5082-5092 (2015).

46. Ramezani-Dakhel H, Mirau PA, Naik RR, Knecht MR, Heinz H. Stability, Surface Features, and Atom Leaching of Palladium Nanoparticles: Toward Prediction of Catalytic Functionality. *Phys Chem Chem Phys* **15**, 5488-5492 (2013).

47. Coppage R, *et al.* Exploiting Localized Surface Binding Effects to Enhance the Catalytic Reactivity of Peptide-Capped Nanoparticles. *J Am Chem Soc* **135**, 11048-11054 (2013).

48. Liu W, Tkatchenko A, Scheffler M. Modeling Adsorption and Reactions of Organic Molecules at Metal Surfaces. *Acc Chem Res* **47**, 3369-3377 (2014).

49. Kawai S, *et al.* Van der Waals Interactions and the Limits of Isolated Atom Models at Interfaces. *Nat Comm* **7**, 11559 (2016).

50. Silvestrelli PL, Ambrosetti A. Van Der Waals Corrected Density Functional Theory Simulation of Adsorption Processes on Noble-Metal Surfaces: Xe on Ag(111), Au(111), and Cu(111). *J Low Temp Phys* **185**, 183-197 (2016).

51. Tao JM, Rappe AM. Physical Adsorption: Theory of van der Waals Interactions between Particles and Clean Surfaces. *Phys Rev Lett* **112**, 106101 (2014).

52. Gross A. *Theoretical Surface Science: A Microscopic Perspective*, 2nd edn. Springer (2009).

53. Yeh IC, Berkowitz ML. Ewald Summation for Systems with Slab Geometry. *J Chem Phys* **111**, 3155-3162 (1999).

54. Lee S, Staehle RW. Adsorption of Water on Gold. *Corrosion* **52**, 843-852 (1996).

55. Wells RL, Fort T. Adsorption of Water on Clean Gold by Measurement of Work Function Changes. *Surf Sci* **32**, 554-560 (1972).

56. Deschanel J-ML. *Adsorption of Water on Gold*. Missouri University of Science and Technology (1973).

57. Heras JM, Albano EV. Adsorption of Water on Gold Films - A Work Function and Thermal-Desorption Mass-Spectrometry Study. *Z Physikalische Chemie-Wiesbaden* **129**, 11-20 (1982).

58. Osman MA, Keller BA. Wettability of Native Silver Surfaces. *Appl Surf Sci* **99**, 261-263 (1996).

59. Gupta A, Boekfa B, Sakurai H, Ehara M, Priyakumar UD. Structure, Interaction, and Dynamics of Au/Pd Bimetallic Nanoalloys Dispersed in Aqueous Ethylpyrrolidone, a Monomeric Moiety of Polyvinylpyrrolidone. *J Phys Chem C* **120**, 17454-17464 (2016).

60. Feng J, Pandey RB, Berry RJ, Farmer BL, Naik RR, Heinz H. Adsorption Mechanism of Single Amino Acid and Surfactant Molecules to Au {111} Surfaces in Aqueous Solution: Design Rules for Metal-Binding Molecules. *Soft Matter* **7**, 2113-2120 (2011).

61. Heinz H, *et al.* Nature of Molecular Interactions of Peptides with Gold, Palladium, and Pd-Au Bimetal Surfaces in Aqueous Solution. *J Am Chem Soc* **131**, 9704-9714 (2009).

62. Ruan L, *et al.* Tailoring Molecular Specificity Toward a Crystal Facet: A Lesson From Biorecognition Toward Pt{111}. *Nano Lett* **13**, 840-846 (2013).

63. Ramezani-Dakhel H, Ruan LY, Huang Y, Heinz H. Molecular Mechanism of Specific Recognition of Cubic Pt Nanocrystals by Peptides and the Concentration-Dependent Formation from Seed Crystals. *Adv Funct Mater* **25**, 1374-1384 (2015).

64. Heinz H, Ramezani-Dakhel H. Simulations of Inorganic–Bioorganic Interfaces to Discover New Materials: Insights, Comparisons to Experiment, Challenges, and Opportunities. *Chem Soc Rev* **45**, 412-448 (2016).

65. Limmer DT, *et al.* Charge Fluctuations in Nanoscale Capacitors. *Phys Rev Lett* **111**, 106102 (2013).

66. Limmer DT, Willard AP. Nanoscale Heterogeneity at the Aqueous Electrolyte-Electrode Interface. *Chem Phys Lett* **620**, 144-150 (2015).

67. Feng J, Slocik JM, Sarikaya M, Naik RR, Farmer BL, Heinz H. Influence of the Shape of Nanostructured Metal Surfaces on Adsorption of Single Peptide Molecules in Aqueous Solution. *Small* **8**, 1049-1059 (2012).
