# Supplementary material for: Insight into induced charges at metal surfaces and biointerfaces using a polarizable Lennard–Jones potential
Source: Nat Commun. 2018 Feb 19;9:716. doi: 10.1038/s41467-018-03137-8 (PMC5818522; doi:10.1038/s41467-018-03137-8)
Supplement: Supplementary file 2 — Description of Additional Supplementary Files [file 41467_2018_3137_MOESM2_ESM.pdf]

Description of Additional Supplementary Files

File Name: Supplementary Movie 1
Description: Dynamics of three isolated physisorbed water molecules on a polarizable Aue (100) surface for 100 ps. The oxygen atoms in water prefer epitaxial (hollow) sites over top sites.

File Name: Supplementary Movie 2
Description: Dynamics of three isolated physisorbed water molecules on a non-polarizable Au (100) surface for 100 ps. The neat Lennard-Jones potential from ref. 10 was used. Some water molecules randomly associate to form hydrogen bonds. The dynamics is very similar to the Au (100) surface with dummy electrons.

File Name: Supplementary Movie 3
Description: Dynamics of three isolated physisorbed water molecules on a polarizable Aue (111) surface for 100 ps. The oxygen atoms in water prefer epitaxial (hollow) sites over top sites. Association of water molecules via hydrogen bonds is seen toward the end.

File Name: Supplementary Movie 4
Description: Dynamics of three isolated physisorbed water molecules on a non-polarizable Au (111) surface for 100 ps. The neat Lennard-Jones potential from ref. 10 was used. The dynamics is very similar to the model with dummy electrons.

File Name: Supplementary Movie 5
Description: Dynamics of a water monolayer on a polarizable Aue (100) surface for 100 ps. A tendency toward square order of the water layer on the epitaxial sites is seen, stabilized by hydrogen bonds between neighbor water molecules.

File Name: Supplementary Movie 6
Description: Dynamics of a water monolayer on a non-polarizable Au (100) surface for 100 ps. The neat Lennard-Jones potential from ref. 10 was used. Similar square order and hydrogen bonds between neighbor water molecules as on the Aue (100) surface with dummy electrons are seen.

File Name: Supplementary Movie 7
Description: Dynamics of a water monolayer on a polarizable Aue (111) surface for 100 ps. The order of water molecules is incommensurate with the epitaxial pattern on the surface and higher mobility of the water molecules relative to the (100) surface is seen.

File Name: Supplementary Movie 8
Description: Dynamics of a water monolayer on a non-polarizable Au (111) surface for 100 ps. The neat Lennard-Jones potential from ref. 10 was used. The observations are very similar to the polarizable Aue (111) model.

File Name: Supplementary Data 1
Description: Supplementary Dataset 1 contains all-atom models to build gold (111), (100), and (110) surfaces in car/mdf format as well as force field files in charmm-27-iff and cvff-iff format ready to run simulations of gold-biological and gold-materials interfaces.
